# Forced expression of miR-143 and -145 in cardiomyocytes induces cardiomyopathy with a reductive redox shift

**DOI:** 10.1186/s11658-020-00232-x

**Published:** 2020-08-24

**Authors:** Kota Ogawa, Akiko Noda, Jun Ueda, Takehiro Ogata, Rumiko Matsuyama, Yuji Nishizawa, Shanlou Qiao, Satoru Iwata, Morihiro Ito, Yoshitaka Fujihara, Masatoshi Ichihara, Koichi Adachi, Yuji Takaoka, Takashi Iwamoto

**Affiliations:** 1 Department of Biomedical Sciences, Chubu University Graduate School of Life and Health Sciences, Kasugai, Aichi Japan; 2 Center for Education in Laboratory Animal Research, Chubu University, Kasugai, Aichi Japan; 3 Present address: Center for Advanced Research and Education, Asahikawa Medical University, Asahikawa, Hokkaido Japan; 4 Department of Pathology and Cell Regulation, Graduate School of Medical Sciences, Kyoto Prefectural University of Medicine, Kyoto, Japan; 5 College of Bioscience and Biotechnology, Chubu University, Kasugai, Aichi Japan; 6 Research Institute for Microbial Diseases, Osaka University, Osaka, Japan; 7 Present address: Department of Bioscience and Genetics, National Cerebral and Cardiovascular Center, Osaka, Japan; 8 Radioisotope Research Center Medical Division, Nagoya University Graduate School of Medicine, Nagoya, Aichi Japan

**Keywords:** Reductive stress, microRNA, Cardiomyopathy, G6PD, p62/SQSTM1, JNK, IRE1α

## Abstract

**Background:**

Animal model studies show that reductive stress is involved in cardiomyopathy and myopathy, but the exact physiological relevance remains unknown. In addition, the microRNAs miR-143 and miR-145 have been shown to be upregulated in cardiac diseases, but the underlying mechanisms associated with these regulators have yet to be explored.

**Methods:**

We developed transgenic mouse lines expressing exogenous miR-143 and miR-145 under the control of the alpha-myosin heavy chain (αMHC) promoter/enhancer.

**Results:**

The two transgenic lines showed dilated cardiomyopathy-like characteristics and early lethality with markedly increased expression of miR-143. The expression of hexokinase 2 (HK2), a cardioprotective gene that is a target of miR-143, was strongly suppressed in the transgenic hearts, but the in vitro HK activity and adenosine triphosphate (ATP) content were comparable to those observed in wild-type mice. In addition, transgenic complementation of HK2 expression did not reduce mortality rates. Although HK2 is crucial for the pentose phosphate pathway (PPP) and glycolysis, the ratio of reduced glutathione (GSH) to oxidized glutathione (GSSG) was unexpectedly higher in the hearts of transgenic mice. The expression of gamma-glutamylcysteine synthetase heavy subunit (γ-GCSc) and the in vitro activity of glutathione reductase (GR) were also higher, suggesting that the recycling of GSH and its de novo biosynthesis were augmented in transgenic hearts. Furthermore, the expression levels of glucose-6-phosphate dehydrogenase (G6PD, a rate-limiting enzyme for the PPP) and p62/SQSTM1 (a potent inducer of glycolysis and glutathione production) were elevated, while p62/SQSTM1 was upregulated at the mRNA level rather than as a result of autophagy inhibition. Consistent with this observation, nuclear factor erythroid-2 related factor 2 (Nrf2), Jun N-terminal kinase (JNK) and inositol-requiring enzyme 1 alpha (IRE1α) were activated, all of which are known to induce p62/SQSTM1 expression.

**Conclusions:**

Overexpression of miR-143 and miR-145 leads to a unique dilated cardiomyopathy phenotype with a reductive redox shift despite marked downregulation of HK2 expression. Reductive stress may be involved in a wider range of cardiomyopathies than previously thought.

## Introduction

The microRNAs miR-143 and miR-145 are located approximately 1.7 kb apart on a bicistronic primary transcript and are strongly co-expressed in smooth muscle cells [1–3]. Their expression is lower in cardiac cells [1], but recent studies have shown that they are both involved in cardiac development and pathophysiology [4–6]. Matkovich et al. demonstrated that miR-143 levels are higher in myocardial samples from patients with cardiomyopathy [4]. In addition, circulating miR-143 levels were found to be significantly higher in the serum of children with dilated cardiomyopathy [7] and miR-145 levels were reportedly higher in the plasma of lamin A/C-related dilated cardiomyopathy patients [8].

We investigated whether the dysregulation of miR-143 and miR-145 is involved in the pathogeneses of cardiac disorders. Using the alpha-myosin heavy chain (αMHC) promoter/enhancer, we developed three lines of transgenic mice that overexpressed both miRNAs, although the levels of miR-143 were significantly higher than those of miR-145. The mice in two of the lines exhibited cardiomyopathy and died at an early age.

The mortality and morbidity of our transgenic mice significantly correlated with the miR-143 expression level. We examined the expressions of miR-143 targets that are known to be involved in cardiomyopathy or cardiac remodeling and observed that hexokinase 2 (HK2) expression was drastically lower in the transgenic hearts.

The miR-143 target HK2 [9] is a glycolytic rate-limiting enzyme that phosphorylates glucose to produce glucose-6-phosphate (G6P). Increasing evidence indicates that it plays a significant role in cardiac function [10–13]. Although the expression of HK2 was drastically suppressed in our transgenic mice, the results of an in vitro HK assay indicated a lack of significant suppression of HK activity. Consistent with this observation, forced expression of the *HK2* gene in the transgenic hearts did not improve mouse survival.

In addition to participating in glycolysis, HK2 plays a crucial role in the pentose phosphate pathway (PPP) [10]. A previous study on heterozygous HK2-deficient mice revealed that downregulation of HK2 expression in the heart promotes cardiac hypertrophy in response to pressure overload by increasing reactive oxygen species (ROS) production [12]. Nevertheless, glutathione production and the ratio of reduced glutathione (GSH) to oxidized disulfide glutathione (GSSG) unexpectedly increased in the hearts of transgenic mice compared with those of control mice in our study.

A shift in the redox state towards oxidative stress is recognized as a leading cause of pathophysiological processes [14]. However, the participation of reductive stress in human disorders and animal disease models was also recently demonstrated [14–17]. Animal cardiomyopathy and myopathy model studies particularly highlighted an association of reductive stress with an aggregation of mutant proteins [18, 19]. These studies have demonstrated the essential roles of G6P dehydrogenase (G6PD), a rate-limiting enzyme for the PPP, and p62, which has recently been shown to induce glutathione production and glycolysis [20, 21], in the pathogeneses of cardiomyopathy and myopathy. Interestingly, the expressions of both molecules were higher in transgenic mice in our study. Although we do not indicate here that reductive stress is the actual cause of cardiomyopathy, our data suggest the involvement of a reductive redox shift in its development.

p62, also referred to as SQSTM1, is an autophagy cargo receptor for the degradation of ubiquitinated substrates that can accumulate upon inhibition of autophagy [22, 23]. It is transcriptionally activated by a variety of signaling processes. For example, activation of Jun N-terminal kinase (JNK) was shown to induce p62 mRNA expression [24, 25]. In addition, two groups recently reported that inositol-requiring enzyme 1 alpha (IRE1α)/JNK signaling associated with endoplasmic reticulum (ER) stress augments p62 expression [26, 27].

Nuclear factor erythroid-2 related factor 2 (Nrf2) is a master transcriptional regulator that controls the basal and inducible expression of a number of antioxidant genes and other cytoprotective phase II detoxifying enzymes [28]. Nrf2 has also been shown to upregulate the expression of p62 [29]. Notably, phosphorylation of p62 dramatically enhances the binding affinity of p62 for Keap1, thereby playing an important role in the stabilization of the Nrf2 protein [30]. Here, Nrf2 expression and JNK, IRE1α and p62 phosphorylation were observed to be enhanced in the hearts of transgenic mice, suggesting that the JNK/IRE1α/p62/Nrf2 signaling cascade is involved in the reductive state.

## Materials and methods

### Plasmid DNA construction

**Fig. 1:**
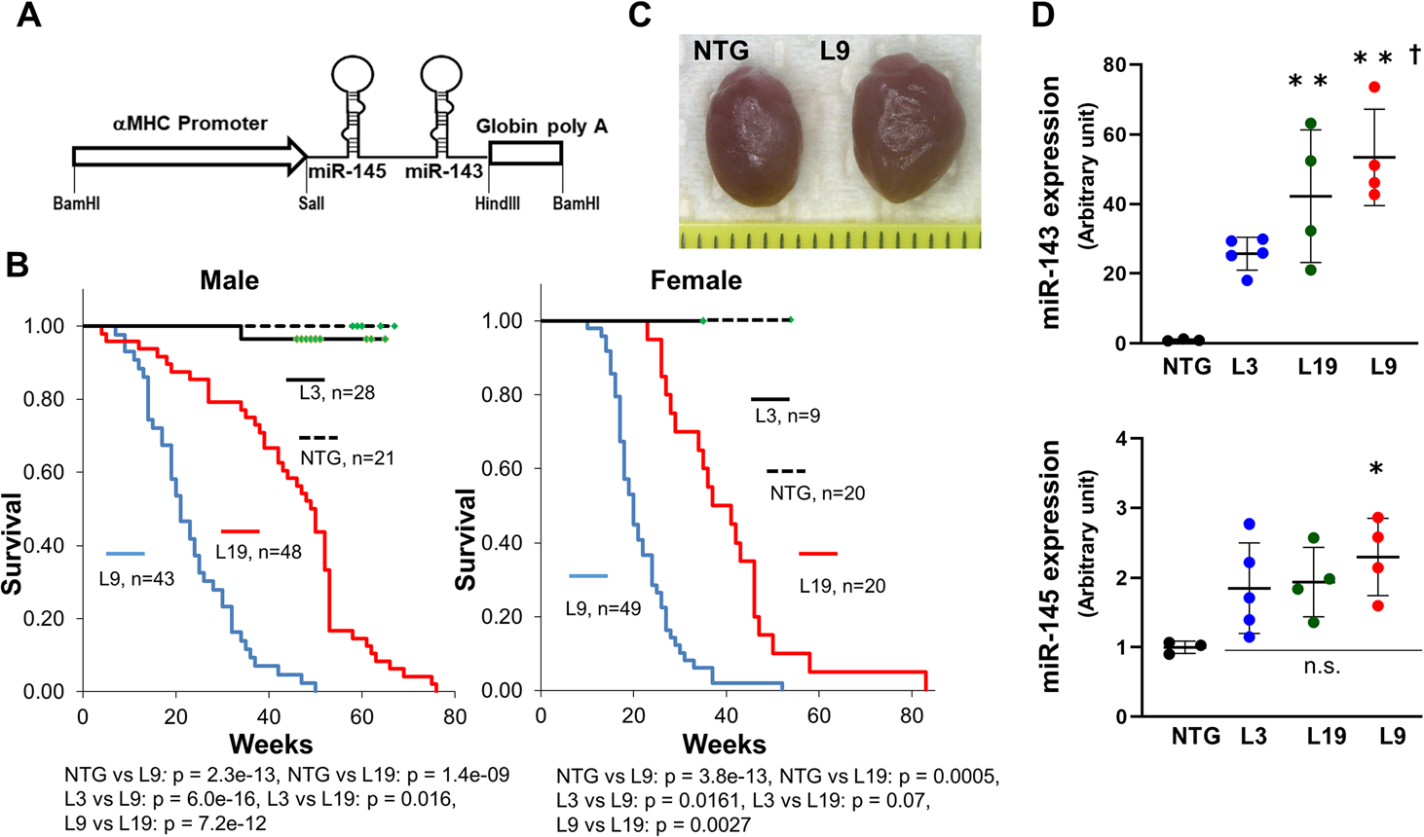
Establishment of αMHC/miR-143/145TG mice. **a** Structure of the injected fragment. An approximately 6.7-kb *Bam*HI fragment containing the pri-miR-145 and pri-miR-143 genes was used. **b** Kaplan-Meier survival analysis of αMHC/miR-143/145TG mice. Data were analyzed using a log-rank test followed by a post hoc Holm test. Green squares = censored. **c** Hearts from representative 5-month old male NTG and L9 mice. The scale bar is 1 mm. **d** Quantitative RT-PCR analysis of miR-143 and miR-145 in the hearts of 3-month old male αMHC/miR-143/145TG mice. The results are presented as the means ± SD with scattered blots. Significance was assessed with one-way ANOVA followed by a post hoc Tukey test (*n* = 3 ~ 5. *p < 0.05 vs. NTG; **p < 0.01 vs. NTG; ^**†**^*p* < 0.05 vs. L3). Similar results were obtained in at least two independent experiments

To construct the plasmid DNA for the transgenic αMHC/miR-143/145TG and αMHC/miR-145TG mice, ~ 650- and ~ 300-base pair (bp) fragments containing the human pri-miR-143 and pri-miR-145 genes or the human pri-miR-145 gene alone were separately subcloned into the *Sal*I and *Hind*III sites of the αMHC promoter/enhancer vector [31]. To construct the plasmid DNA for αMHC/HK2TG mice, the full-length human HK2 cDNA fragment from FLHKII-pGFPN3 (#21920; Addgene, USA) [32] was subcloned into the *Sal*I and *Hind*III sites of the αMHC promoter/enhancer vector. Subsequently, *Bam*HI-*Bam*HI fragments of αMHC/miR-143/145TG, αMHC/miR-145TG and αMHC/HK2TG mice were purified from agarose gels with ELUTIP-D (#10462617; GE Healthcare, USA), and injected into mouse oocytes (Fig. 1a).

### Animal experiments

The mice were housed in an environment with constant temperature (22 ± 2 °C) and humidity (50 ± 10%). They had a 12 h light/12 h dark cycle and free access to water and food.

To establish the transgenic mice (αMHC/miR-143/145TG, αMHC/miR-145TG and αMHC/HK2TG), B6D2F1 females (C57BL/6 N female × DBA/2 male) were mated with B6D2F1 males the night before egg injection. The eggs were prepared for injection as described previously [33] and implanted into the oviducts of the pseudo-pregnant ICR mice the next day. All surgeries were performed under anesthesia by intraperitoneal injection of a mixture of three drugs: 0.75 mg/kg medetomidine (Nippon Zenyaku Kogyo, Japan), 4 mg/kg midazolam (Sandoz, USA), and 5 mg/kg butorphanol (Meiji Seika Pharma, Japan). The transgenic progeny were backcrossed to C57BL/6 J mice at least four times before the experiments. Primers for checking transgene transmission are shown in Additional file 1. For the echocardiography, mice were anesthetized with 1.5% isoflurane (Escain; Mylan, USA) through a Narcobit-E inhalation anesthesia apparatus (Natsume Seisakusho, Japan).

For αMHC/miR-145TG mice, the injections were performed at the Research Center for Molecular Genetics, Institute for Promotion of Medical Science Research, Yamagata University Faculty of Medicine. The injection for αMHC/HK2TG were done at the Research Institute for Microbial Diseases, Osaka University. All other animal experiments were performed at the Center for Education in Laboratory Animal Research, Chubu University.

### Western analysis

The heart tissue was frozen in liquid nitrogen. It was homogenized using a TissueLyser (Qiagen, Germany) at 1500 rpm for 2 min in cold RIPA buffer, which consisted of 10 mM Tris-HCl (pH 7.4), 150 mM NaCl, 5 mM ethylenediaminetetraacetic acid (EDTA), 1% Triton-X, 0.1% sodium dodecyl sulfate and 1% sodium deoxycholate, together with 1 mM benzylsulfonyl fluoride, 0.01 M NaF, 2 mM sodium orthovanadate and a protease inhibitor cocktail (#25955–24; Nacalai Tesque, Japan). After centrifugation at 12,000×g and 4 °C for 15 min, the protein concentration of the supernatant was determined with a Pierce® BCA Protein Assay kit (#23227; Thermo Fisher Scientific, USA).

Then, protein samples were incubated in 1× Laemmli Sample Buffer (#1610737; Bio-Rad Laboratories, USA) with 0.358 M mercaptoethanol at 98 °C for 10 min and sonicated with a SONIFIER 250 (Branson Ultrasonics Corporation, USA). Subsequently, 25–50 μg of protein was electrophoresed on SDS-polyacrylamide gels and transferred to a PVDF membrane (Immobilon-P®; Merck Millipore, USA). The membrane was blocked with 5% skim milk in phosphate-buffered saline (PBS) solution with 0.05% Tween 20 at 37 °C for 30 min, incubated with the various antibodies according to the manufacturers’ instructions, and visualized using Pierce™ ECL Western Blotting Substrate (#32106; Thermo Fisher Scientific, USA). The membrane was analyzed with FUSION-SOLO.4S (Vilber Lourmat, France), densitometric analysis was performed with Fusion Capture, and GAPDH was used for normalization. The Y axes of the densitometric analysis graphs present the ratio as an arbitrary unit. The antibodies are shown in Additional file 2.

### Quantitative reverse transcription PCR

Heart tissue in TRI Reagent® (#TR118; Molecular Research Center, USA) was homogenized using a TissueLyser (Qiagen, Germany) at 1500 rpm for 2 min, and total RNA was extracted according to the manufacturer’s instructions. Standard quantitative reverse transcription PCR (RT-PCR) was performed with SYBR Green as the dye. Briefly, one μg of total RNA was used for reverse transcription with PrimeScript™ RT Master Mix (#RR036A; Takara Bio, Japan), after which quantitative PCR was performed using KOD SYBR® qPCR Mix (#QKD-201; Toyobo, Japan) on a CFX96 Touch Real-Time PCR Detection System (Bio-Rad Laboratories, USA). All values were corrected by each calibration curve, and the relative expression level was measured with the ΔΔCt method using the *Ywhaz* gene for normalization. Primers for quantitative RT-PCR are shown in Additional file 1.

To assess the expression of mature miR-143 and miR-145, reverse transcription was performed with a Taqman® MicroRNA Reverse Transcription kit (#4366597; Thermo Fisher Scientific, USA), and quantitative PCR was performed with Premix EX Taq™ (#RR390A; Takara Bio, Japan) using Taqman® MicroRNA Assays (#4427975; Thermo Fisher Scientific, USA). The primers used were hsa-miR-143 (P/N: 002249) and hsa-miR-145 (P/N: 002278), and snoRNA202 (P/N: 001232; all from Thermo Fisher Scientific, USA) was used for normalization.

### Hexokinase assay

Hexokinase activity™ was calculated with a Colorimetric Hexokinase Activity Assay kit (#ab136957; Abcam, UK) according to the manufacturer’s instructions. Briefly, the heart tissue was frozen in liquid nitrogen, mixed with cold assay buffer and then homogenized using a TissueLyser (Qiagen, Germany) at 1500 rpm for 1 min. The samples were centrifuged at 4 °C for 5 min at 12,000×g, and the supernatant was diluted 100-fold with assay buffer. Then, the samples were analyzed via absorption spectrophotometry using a Sunrise™ spectrophotometer (Tecan Group, Switzerland), with the absorbance at 450 nm recorded every 3 min for 30 min in kinetic mode. The values were corrected with protein concentration.

### ATP assay

The content of adenosine triphosphate (ATP) was calculated with an AMERIC-ATP (T) kit (#632–23,881; FUJIFILM Wako Pure Chemical Corporation, Japan) according to the manufacturer’s instructions. Briefly, the heart tissue was frozen in liquid nitrogen, mixed with cold extraction buffer A and then homogenized using a TissueLyser (Qiagen, Germany) at 1500 rpm for 2 min. After the addition of extraction buffer B, the samples were vortexed and then centrifuged at 4 °C for 5 min at 10,000×g. Then, the supernatant was diluted 2000-fold with reaction buffer, mixed with luciferase reaction mixture, and analyzed using a Luminometer (Lumat LB 9507; Berthold Technologies, Germany). The values were corrected with the tissue wet weight.

### TBARS assay

Ethanol with 5% 2,6-di-t-butyl-p-cresol (BHT, #11421–92; Nacalai Tesque, Japan) was diluted with PBS to generate a lysis buffer containing 0.05% BHT. The heart tissue was frozen via liquid nitrogen, mixed with cold lysis buffer (10 μl/mg), and then homogenized using a TissueLyser (Qiagen, Germany) at 1500 rpm for 1 min. After centrifugation at 12,000×g at 4 °C for 5 min, 50 μl of supernatant was collected, the same amount of 1.8% SDS solution was added and the mixture was vortexed and allowed to stand for 5 min. Subsequently, 125 μl of a 2-thiobarbituric acid (TBA, #T5500; Sigma-Aldrich, USA) solution (2.5 ml of 21% acetic acid, 272 μl of 5 M NaOH, 250 μl of distilled water) was added, and the resulting solution was vortexed, incubated at 95 °C for 50 min, stored on ice for 5 min, and then centrifuged at 3000 rpm at room temperature for 15 min. The supernatant was then analyzed via absorption spectrophotometry with an Ultrospec 3100 pro (GE Healthcare, USA), and the absorbance at 532 nm was recorded. 1,1,3,3-tetramethoxypropane (MDA, # 206–08962; FUJIFILM Wako Pure Chemical Corporation, Japan) was used as a standard. The values of the samples were corrected with the protein concentrations.

### GSH/GSSG assay

The assay was performed using a GSSG/GSH Quantification kit (#G257; DOJINDO Laboratories, Japan) according to the manufacturer’s instructions. Briefly, the heart tissue was frozen in liquid nitrogen, mixed with cold lysis buffer (5% 5-sulfosalicylic acid dihydrate: #197–04582; FUJIFILM Wako Pure Chemical Corporation, Japan), and then homogenized using a TissueLyser (Qiagen, Germany) at 1500 rpm for 2 min. After centrifugation at 8000×g at 4 °C for 10 min, the supernatant was collected and analyzed by measuring the absorbance at 405 nm with a Sunrise™ spectrophotometer (Tecan Group, Switzerland), and the values were corrected with the tissue wet weight.

### Reduced nicotinamide adenine dinucleotide phosphate (NADPH)/NADP^+^ assay

The contents of NADPH and NADP^+^ were calculated with a NADP^+^/NADPH Assay kit (#MET-5018; Cell Biolabs, USA) with slight modification. Briefly, the heart tissue was frozen in liquid nitrogen, mixed with cold 1× extraction buffer, and then homogenized using a TissueLyser (Qiagen, Germany) at 1500 rpm for 2 min. After centrifugation at 15,000 rpm and 4 °C for 5 min, the supernatant was filtered with a 10 kDa spin filter (#OD010C33; Pall Corporation, USA). To measure NADPH and NADP^+^ contents, 25 μl of the flow through was mixed with 5 μl of 0.1 N NaOH and 0.1 N HCl, respectively, and then incubated at 80 °C for 1 h. After the addition of 20 μl of 1× assay buffer, the samples were mixed with 50 μl of working solution (#N510; DOJINDO Laboratories, Japan) and then analyzed. The absorbance of the samples at 450 nm was recorded with a Sunrise™ spectrophotometer (Tecan Group, Switzerland), and the values were corrected with the tissue wet weight.

### Glutathione reductase assay

Glutathione reductase (GR) activity was determined with a Glutathione Reductase Assay kit (#STA-812; Cell Biolabs, USA) according to the manufacturer’s instructions. Briefly, the heart tissue was frozen in liquid nitrogen, mixed with cold PBS/1 mM EDTA and then homogenized using a TissueLyser (Qiagen, Germany) at 1500 rpm for 2 min. After centrifugation at 15,000 rpm at 4 °C for 5 min, the supernatant was diluted 20-fold with 1× assay buffer, then mixed with 1× NADPH solution, 1× Chromogen and GSSG solution. After brief mixing, the samples were analyzed via absorption spectrophotometry using a Sunrise™ spectrophotometer (Tecan Group, Switzerland) with the absorbance at 405 nm recorded every 1 min for 12 min in kinetic mode. The values were corrected with the tissue wet weight.

### Echocardiography

Mice were imaged using a Xario instrument with a 12-MHz linear probe (PLT-1202S; Canon Medical Systems, Japan), and ECG monitoring was performed using limb electrodes. Interventricular septal thickness (IVST), LV posterior wall thickness (LVPWT), and LV end-diastolic and end-systolic diameters (LVDd and LVDs) were obtained from a short-axis view. Percent LV fractional shortening (%LVFS) was calculated as an index of LV systolic function, and LV mass was measured to assess LV hypertrophy.

### cDNA microarray analysis

Total RNA of 3-month old L9, L19 and control NTG hearts was extracted with TRI Reagent® and then assayed with a Toray 3D-Gene Mouse Oligo chip 24 (Toray Industries, Japan). Briefly, total RNA was labeled with Cy5 or Cy3 using an Amino Allyl MessageAMP II aRNA Amplification kit (#AM1753; Thermo Fisher Scientific, USA). The Cy5- or Cy3-labeled aRNA pools were mixed with hybridization buffer and hybridized for 16 h according to the manufacturer’s protocols (www.3d-gene.com). The hybridization signals were obtained using a 3D-Gene Scanner and processed with 3D-Gene Extraction software (Toray Industries, Japan). The detected signals for each gene were normalized using the global normalization method (Cy3 to Cy5 ratio median = 1). Differential gene expression was determined based on a fold change cutoff of > 1.5 compared to the average for the NTG control mice. A heatmap was generated using Java Tree View (http://jtreeview.sourceforge.net). Genes that were differentially expressed at least 1.5-fold in L9 and L19 mice compared to the NTG control were identified and ontologically classified using Ingenuity Pathway Analysis (Qiagen Bioinformatics, USA). Significant associations with the functional categories were identified using Fisher’s exact test with a *p*-value cutoff of 0.05.

### Statistical analysis

Two-tailed unpaired t-tests, Fisher’s exact tests or one-way analysis of variance (ANOVA) followed by post hoc Tukey’s multiple comparison tests were performed as described in the figure legends. Kaplan*-*Meier survival was evaluated with log-rank test followed by Holm’s adjustment. All statistical analyses were carried out using EZR [34]. The results are presented as the means ± standard deviation (SD). *p* < 0.05 was considered statistically significant.

## Results

### Establishment of αMHC/miR-143/145TG mice

To express miR-143 and miR-145 simultaneously in cardiomyocytes, we established the 3 lines of transgenic mice (αMHC/miR-143/145TG mice: L3, L9, and L19). The L9 and L19 mice died significantly earlier than their non-transgenic (NTG) littermates (Fig. 1b). The L9 mice showed gradual cardiac enlargement followed by death starting at an approximate age of 8 weeks (Fig. 1c). Of the 28 male and 9 female L3 mice, only one male died during the 34 weeks of observation.

We examined the expression of miR-143 and miR-145 in the transgenic hearts via quantitative RT-PCR analysis. The expression of miR-143 in L9 hearts was over 40 times higher than that observed in NTG hearts and approximately 2 times higher than that detected in L3 hearts (Fig. 1d). By contrast, the expression of miR-145 in transgenic hearts was only approximately 2 times higher than that detected in NTG hearts, with no significant differences observed among the three transgenic lines (Fig. 1d). These findings indicate that mortality is closely associated with the expression level of miR-143. As no clear difference in mortality between male and female mice was observed, we focused our investigation on male L9 mice.

### Dilated cardiomyopathy-like features occur in αMHC/miR-143/145TG mice

**Fig. 2:**
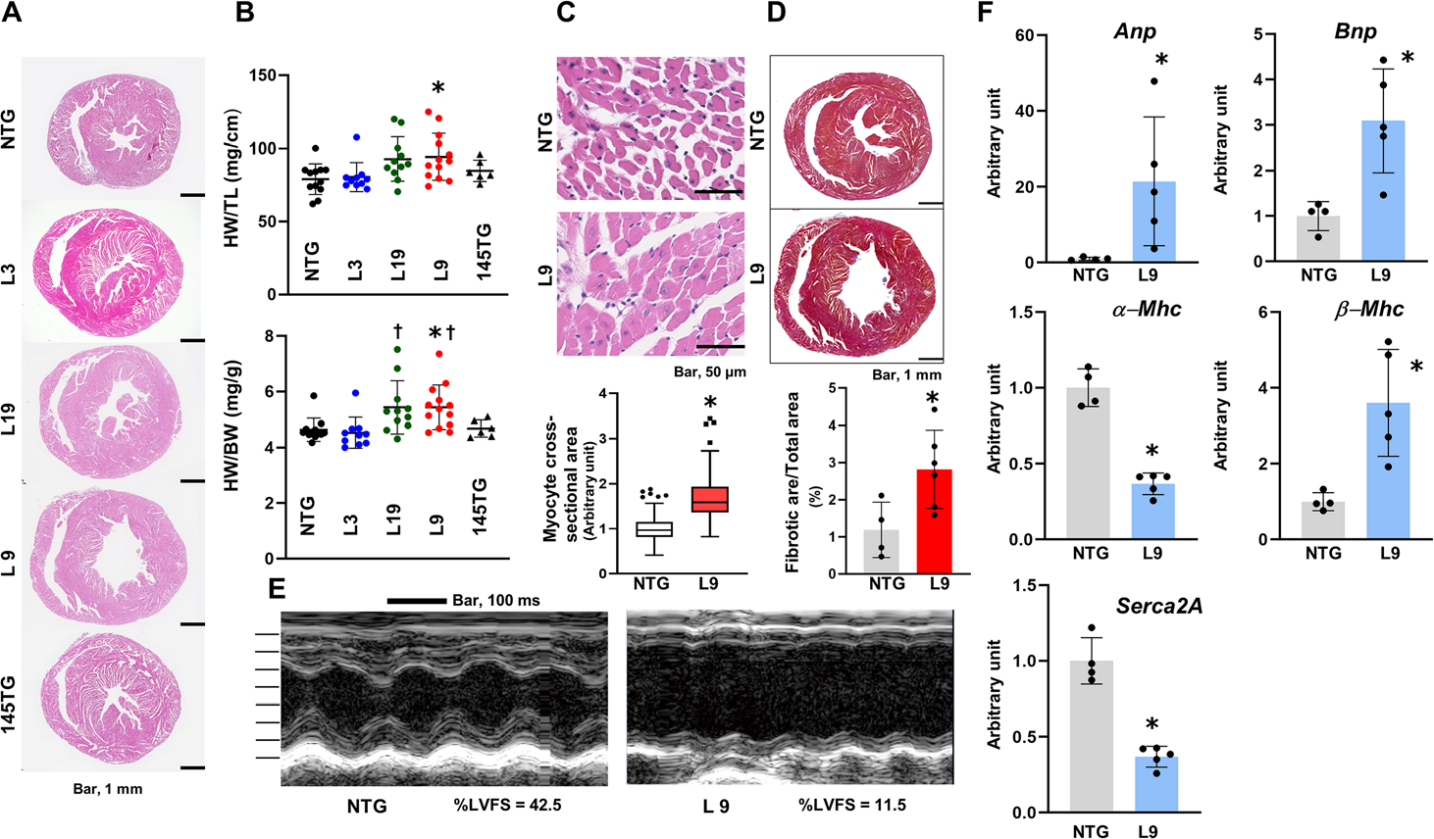
Characterization of the hearts in αMHC/miR-143/145TG and αMHC/miR-145TG mice. **a** Macroscopic histological analysis of hematoxylin & eosin-stained hearts of 4-month old male αMHC/miR-143/145TG and αMHC/miR-145 TG mice. **b** Heart weight corrected for tibia length (upper panel) or body weight (lower panel) of male αMHC/miR-143/145TG and αMHC/miR-145TG mice. The results are presented as the means ± SD with scattered blots. Significance was assessed with one-way ANOVA followed by a post hoc Tukey test (*n* = 6 ~ 13; *p < 0.05 vs. NTG, ^**†**^*p* < 0.05 vs. L3). **c** Microscopic histological analysis of hematoxylin & eosin-stained hearts of 4-month old male L9 mice. The lower panel shows the quantitative analysis of the myocyte cross-sectional area. We analyzed 316 cardiomyocytes of four L9 mice at 4 months of age and 292 cardiomyocytes of three NTG mice. Data are presented as box and whisker plots with the Tukey method and unpaired *t*-test applied (*p < 0.05 vs. NTG). **d** Macroscopic histological analysis of Masson-trichrome-stained hearts of 4-month old male L9 mice. The lower panel shows the quantitative analysis for the fibrotic area. The results are presented as the means ± SD with the unpaired *t*-test applied to determine significance (*n* = 4 ~ 6; *p < 0.05 vs. NTG). **e** Representative M-mode echocardiography of a 3-month old male L9 mouse. The interval of each scale bar of the Y-axis is 1 mm. %LVFS: percentage left ventricular fractional shortening. **f** Quantitative RT-PCR analysis of molecules correlating with cardiac remolding of 3-month old male L9 mice. The results are presented as the means ± SD with the unpaired *t*-test applied to determine significance (*n* = 4 ~ 5; *P < 0.05 vs. NTG). Similar results were obtained in at least two independent experiments

Macroscopic examination revealed enlargement of the left ventricular cavity in 4-month old L9 and L19 mice (Fig. 2a). The hearts of these mice were marginally heavier than those of the NTG and L3 mice (Fig. 2b). However, the hearts of the L3 mice became enlarged after the age of 6 months, and severe enlargement of the left ventricle was observed in the heart of the L3 mouse that died (Additional file 3). The smaller increase in miR-143 expression observed in L3 mice may have delayed the onset of cardiac remodeling in these mice, although observation over a longer period is required to assess this possibility.

We then evaluated the myocyte cross-sectional areas of the L9 hearts and observed that they were 1.7 times larger than those of the NTG hearts, suggesting hypertrophic growth of individual transgenic cardiomyocytes (Fig. 2c). Macroscopic Masson’s trichrome staining results showed that fibrosis was 2.8 times more severe in the hearts of L9 mice than in those of NTG mice (Fig. 2d).

Next, we performed an echocardiographic analysis of the L9 mice. The left ventricular wall motion showed diffuse hypokinesis, and the left ventricular cavity was severely enlarged in the L9 hearts (Fig. 2e). As summarized in Table 1, the left ventricular internal dimensions at both diastole and systole were significantly greater in the L9 hearts than in the NTG hearts, and the increases in these parameters were associated with reductions in posterior wall thickness and interventricular septal thickness. In addition, the percentage of left ventricular fractional shortening (%LVFS), a measure of systolic function, was observed to be greatly reduced in the L9 hearts (Table 1).

**Table 1:** Echocardiographic data for αMHC/miR-143/145TG (L9) mice

	NTG	L9
BW (g)	30.41 ± 1.06	30.04 ± 1.01
HW (mg)	155.33 ± 11.71	156.88 ± 15.96
IVST (mm)	0.62 ± 0.06	0.59 ± 0.06
LVPWT (mm)	0.62 ± 0.16	0.51 ± 0.10*
LVIDd (mm)	3.99 ± 0.24	5.10 ± 0.38**
LVIDs (mm)	2.38 ± 0.28	4.08 ± 0.57**
%LVFS	40.47 ± 4.71	20.32 ± 7.41**

The echocardiographic parameters of 3-month old male αMHC/miR-143/145TG (L9) mice are shown. Unpaired *t*-tests were used (*n* = 8 ~ 9. *p < 0.05 vs. NTG, **p < 0.01 vs. NTG). The data are presented as the means ± standard deviation (SD). BW: body weight, HW: heart weight, IVST: interventricular septal thickness, LVPWT: left ventricular posterior wall thickness, LVIDd: left ventricular internal dimension at diastole, LVIDs: left ventricular internal dimension at systole, %LVFS: percentage of left ventricular fractional shortening.

Quantitative RT-PCR analysis was performed to examine the expression of molecules involved in heart remodeling. Figure 2f shows that the expressions of atrial natriuretic peptide (*Anp),* brain natriuretic peptide (*Bnp)* and β-myosin heavy chain *(β-Mhc)* were higher in L9 hearts. By contrast, the expressions of *α-Mhc* and sarco-endoplasmic reticulum calcium adenosine triphosphatase-2a (*Serca2A*) were lower. These results are consistent with the external appearances and functioning of the transgenic hearts.

For unknown reasons, the expression of miR-145 was much lower than that of miR-143 in all three transgenic mouse lines. To investigate the effects of high cardiac miR-145 expression, we established a line of transgenic mice with the human pri-miR-145 gene under the control of the αMHC promoter/enhancer. These αMHC/miR-145TG mice exhibited 14.8 times higher miR-145 expression than NTG mice (Additional file 4 A and B). However, the appearances and weights of the hearts of these mice were comparable to those of the NTG mice (Fig. 2a and b). All mice (12 males and 9 females) appeared healthy until they reached an age of 9 months. These observations indicate that the high mortality among αMHC/miR-143/145TG mice was primarily due to miR-143 overexpression.

### Marked downregulation of HK2 expression does not play a pivotal role in cardiac pathogenesis

To identify the target molecules of miR-143 that are crucial for the pathogenesis of αMHC/miR-143/145TG mice, we performed cDNA microarray analysis of the hearts of 3-month old L9 and L19 mice. We found that the expressions of HK2 [9, 35, 36] and insulin-like growth factor-binding protein 5 (IGFBP5), a modulator of IGF signaling [37–40], were drastically suppressed compared to the expressions in NTG mice (HK2: 37.5%, IGFBP5: 50.5%; the data were deposited in NCBI GEO under accession number GSE112355). Interestingly, HK2 has been shown to be essential for cardiac function [11–13]. We also analyzed the expressions of extracellular signal-regulated protein kinase 5 (ERK5) [33, 41–44], insulin-like growth factor 1 receptor (IGF1R) [45–48] and oxysterol-binding protein-related protein 8 (ORP8) [49–52], as these molecules have been shown to be targets of miR-143 by at least 4 groups and are involved in cardiomyopathy or cardiac remodeling. In particular, IGF1R has been demonstrated to be a target of both miR-143 and miR-145 [53].

**Fig. 3:**
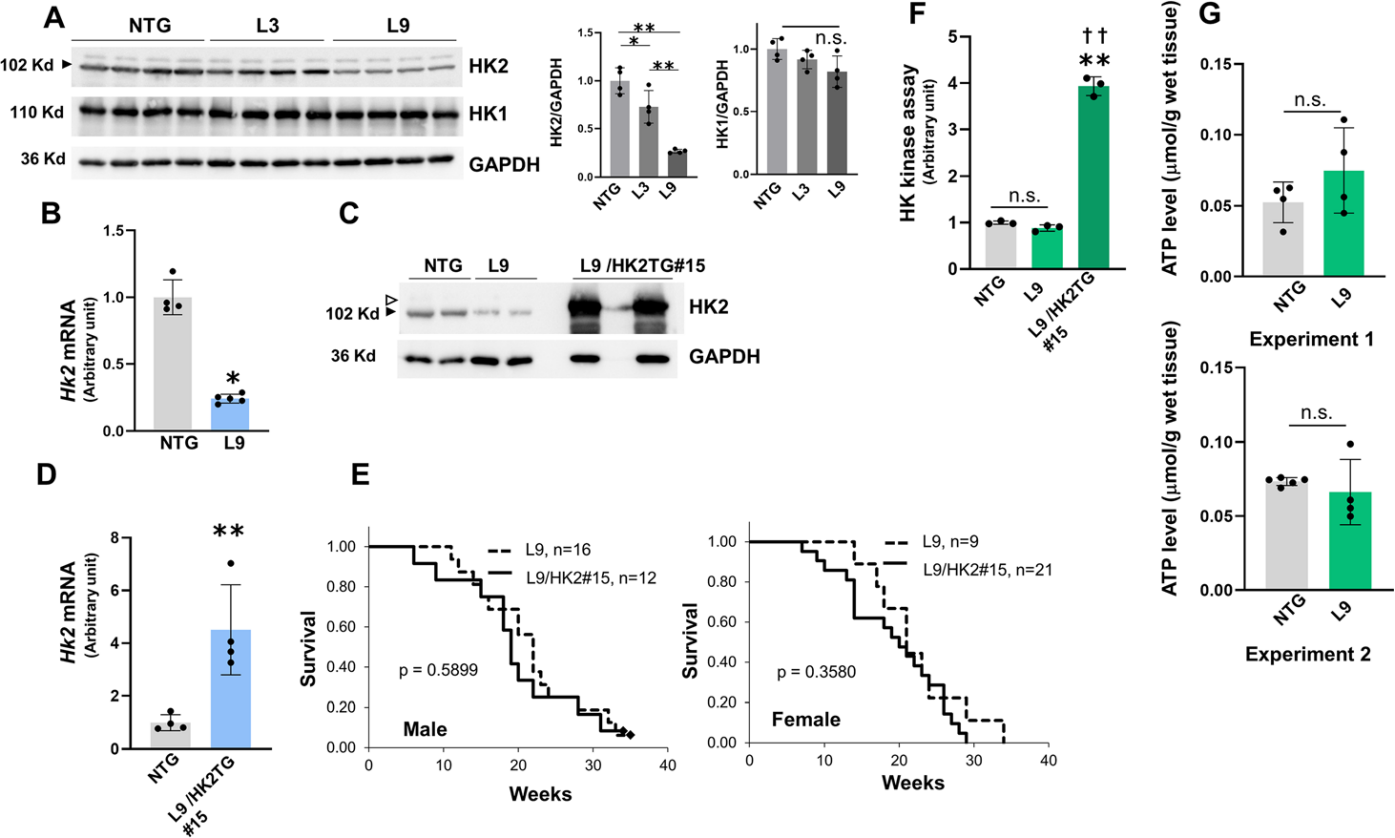
Analysis of HK2 expression, HK activity and ATP content of αMHC/miR-143/145TG (L9) mice and characterization of αMHC/miR-143/145/HK2TG (L9/HK2) mice. **a** Whole cell extracts from the hearts of 3-month old male L3 and L9 mice were examined using the indicated antibodies. The right panels show the relative densitometric analysis of the western blots. The results are presented as the means ± SD. Significance was assessed with one-way ANOVA followed by a post hoc Tukey test (*p < 0.05, **p < 0.01). **b** Quantitative RT-PCR analysis of *Hk2* mRNA in the hearts of 3-month old male L9 mice. The results are presented as the means ± SD with the unpaired *t*-test applied to determine significance (*n* = 4 ~ 5; *p < 0.05 vs. NTG). **c** Western analysis of HK2 in the hearts of 3-month old male L9 and L9/HK2 mice. Whole cell extracts were examined with an anti-HK2 antibody. The white and black arrowheads respectively indicate the transgenic human HK2 and the endogenous mouse HK2. **d** Quantitative RT-PCR analysis of *Hk2* mRNA in the hearts of 3-month old male of L9/HK2 mice. Primers with common binding sites for human and mouse *Hk2* genes were used. The results are presented as the means ± SD with the unpaired *t*-test applied to determine significance (*n* = 4; **p < 0.01 vs. NTG). **e** Kaplan-Meier survival analysis of L9 and L9/HK2 mice. Data were analyzed using log-rank test. **f** Hexokinase assay of the hearts of 3-month old male L9 and L9/HK2 mice. The results are presented as the means ± SD. Significance was assessed with one-way ANOVA followed by a post hoc Tukey test (*n* = 3; **p < 0.01 vs. NTG, ^**††**^*p* < 0.01 vs. L9). **g** ATP content assay in the hearts of 4-week old male L9 mice. The results are presented as the means ± SD with the unpaired *t*-test applied to determine significance (*n* = 4 ~ 5). Experiments 1 and 2 were performed independently. **a–d**, **f** Similar results were obtained in at least two independent experiments

Western analysis showed that HK2 protein levels were drastically lower in αMHC/miR-143/145TG mice than in NTG mice (Fig. 3a, Additional file 5), although the levels of the other assayed proteins were comparable in the L9 and NTG mouse hearts (Additional file 6). In addition, the downregulation of HK2 was 2.7 times greater in L9 hearts than that observed in L3 hearts, and HK2 and miR-143 expression exhibited a certain inverse correlation, indicating that HK2 is a bona fide target of miR-143. We also performed quantitative RT-PCR for *Hk2* mRNA and observed that its expression in L9 hearts was 24% of that detected in NTG hearts (Fig. 3b).

HK1 and HK2 are the primary HK isotypes present in the heart. The expression of HK1 was found to be similar between αMHC/miR-143/145TG and NTG mice (Fig. 3a). Since heterozygous deletion of the *Hk2* gene has been shown to be deleterious for cardiac function [12], we hypothesized that the marked reductions in HK2 protein levels may have been involved in the pathogeneses in these transgenic mice. To elucidate whether *Hk2* gene complementation would attenuate the αMHC/miR-143/145TG phenotype, we established four lines of αMHC/HK2TG mice (#1, #2, #12 and #15) expressing human HK2 in cardiomyocytes (Additional file 7 A). Western analysis revealed that the transgenic human HK2 protein bands were much stronger and slightly larger than the endogenous mouse HK2 bands (Additional file 7 B), in agreement with the results of a previous study [54].

We then crossed αMHC/HK2TG (#1, #2, #15) mice and L9 mice. HK2 was expressed at high levels in the hearts of αMHC/miR-143/145/HK2TG #15 mice (Fig. 3c). We also performed quantitative RT-PCR analysis for *Hk2* mRNA using a pair of primers with sequences common to both the human and mouse *Hk2* genes. *Hk2* mRNA expression in αMHC/miR-143/145/HK2TG mice was approximately 4 times greater than that observed in NTG mice (Fig. 3d). As the HK2 antibody (Additional file 2) used in this study was produced via immunization with human peptides, the considerable difference in protein band intensity between the human transgenic and mouse endogenous HK2 (Fig. 3c) was likely largely due to interspecies differences in antibody affinity towards the HK2 protein.

Notably, the mortality rates of the αMHC/miR-143/145/HK2TG and αMHC/miR-143/145TG mice were similar (Fig. 3e, Additional file 7 C and 7D). Thus, to confirm the activity of transgenic HK2, we performed an in vitro HK assay. The total HK activity in αMHC/miR-143/145TG mice was unexpectedly similar to that observed in NTG mice (Fig. 3f), and this observation is explored in the Discussion section. By contrast, the HK activity in αMHC/miR-143/145/HK2TG mice was approximately 4 times greater than that observed in NTG mice, consistent with the quantitative RT-PCR data for *Hk2* (Fig. 3d).

Next, we examined the ATP content to investigate whether the reduced HK2 protein levels decreased energy production. The ATP content in αMHC/miR-143/145TG hearts was comparable to that observed in NTG hearts (Fig. 3g).

These findings suggest that the striking downregulation of HK2 protein expression would not play a crucial pathogenetic role in αMHC/miR-143/145TG mice.

### Redox balance is shifted towards a reductive state in αMHC/miR-143/145TG mice

**Fig. 4:**
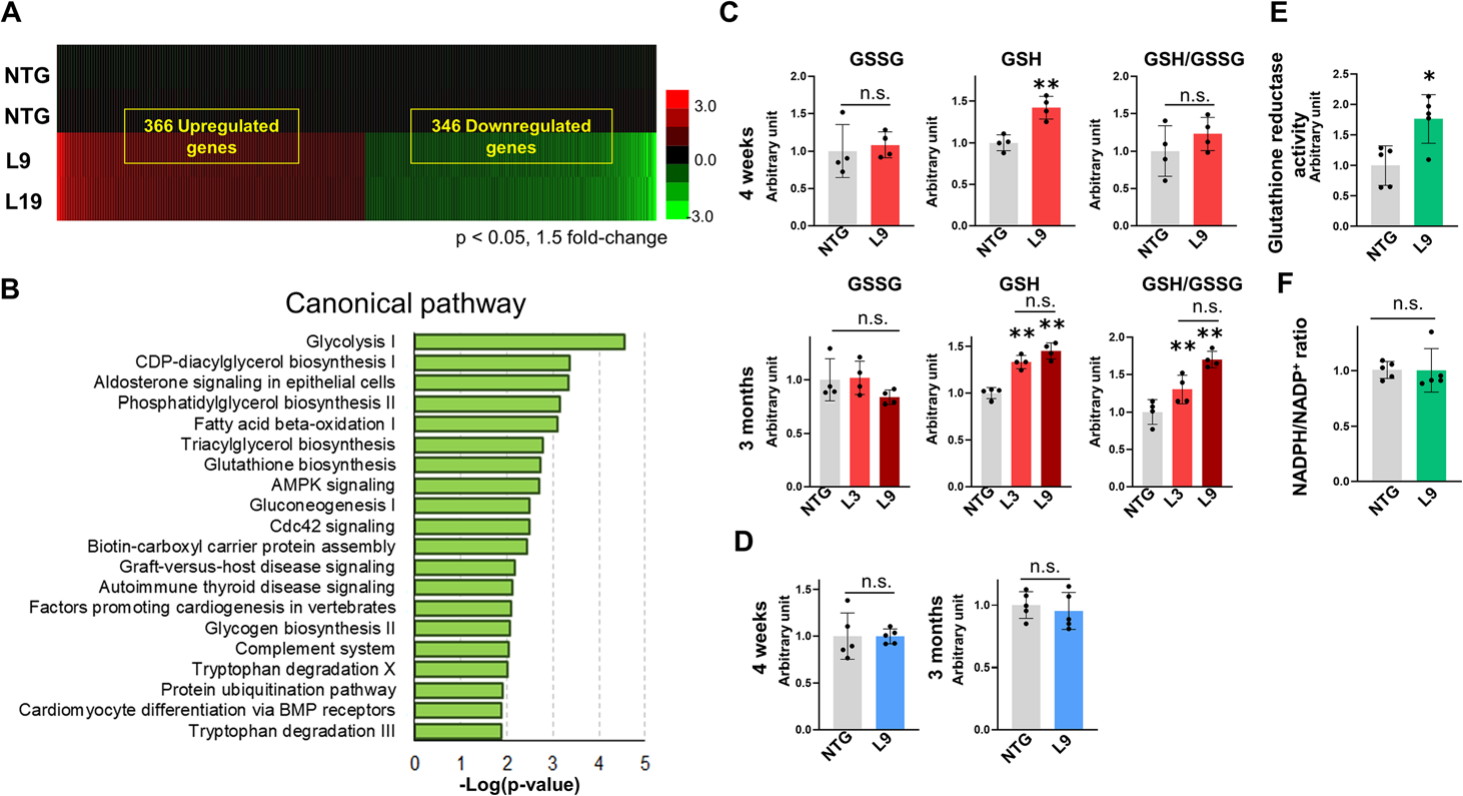
cDNA microarray examination and redox analysis of αMHC/miR-143/145TG mice. **a** Fold changes in gene expression over the average of the male NTG control mice are as indicated on the scale bar, where red indicates upregulation and green indicates downregulation. The number of genes > 1.5-fold differentially expressed in male L9 and L19 mice over control NTG are indicated. **b** Gene ontology classification of genes differentially expressed at least 1.5-fold in male L9 and L19 mice compared to the NTG control. The top twenty most significant canonical pathways in L9 and L19 mice are shown. **c** GSSG, GSH and GSH-to-GSSG ratio of 4-week and 3-month old male L3 and L9 mice. The results are presented as the means ± SD. Significance was assessed with unpaired *t*-test or one-way ANOVA followed by a post hoc Tukey test (*n* = 4; **p < 0.01 vs. NTG). **d** TBARS assay of 4-week and 3-month old male L9 mice. The results are presented as the means ± SD. Significance was assessed with unpaired *t*-test (*n* = 5). **e** Glutathione reductase assay of 3-month-old male L9 mice. The results are presented as the means ± SD. Significance was assessed with unpaired *t*-test (*n* = 5). **f** NADPH/NADP^+^ assay of 3-month old male L9 mice. The ratios of NADPH to NADP^+^ are shown. The results are presented as the means ± SD. Significance was assessed with unpaired *t*-test (*n* = 5). **c–f** Similar results were obtained in at least two independent experiments

To investigate the global changes in mRNA expression in the transgenic mouse hearts, we performed gene ontology (GO) enrichment analysis of cDNA microarray data (Fig. 4a and b). Intriguingly, genes associated with glucose metabolism and glutathione biosynthesis, processes which deeply involve HK2, were expressed at significantly higher levels in transgenic mouse hearts than in NTG mouse hearts.

We then analyzed the production of GSH and GSSG to evaluate the redox state. GSH production and the GSH-to-GSSG ratio were increased in the hearts of 3-month old L3 and L9 mice, clearly indicating that a reductive redox shift occurred in the transgenic hearts (Fig. 4c). These findings were surprising, as a plethora of studies have revealed that oxidative stress is a major factor in the progression of cardiomyopathy [55].

To evaluate ROS production, we used a thiobarbituric acid reactive substances (TBARS) assay to measure the levels of lipid peroxidation products. The results revealed no significant differences in malondialdehyde production between the L9 and NTG mice (Fig. 4d).

These findings suggest that miR-143 overexpression leads to a reductive rather than an oxidative redox state.

### Evidence of elevated recycling and de novo biosynthesis of GSH in transgenic hearts

We were interested in the mechanism behind the reductive redox shift observed in αMHC/miR-143/145TG hearts. GSH systems use NADPH as a source of reducing equivalents, and GSH is generated by recycling GSSG via the oxidation of NADPH through GR. We performed an in vitro GR activity assay. GR activity was 1.76 times higher in L9 hearts than that observed in NTG mouse hearts (Fig. 4e), suggesting that the production of GSH was accelerated via the recycling pathway.

**Fig. 5:**
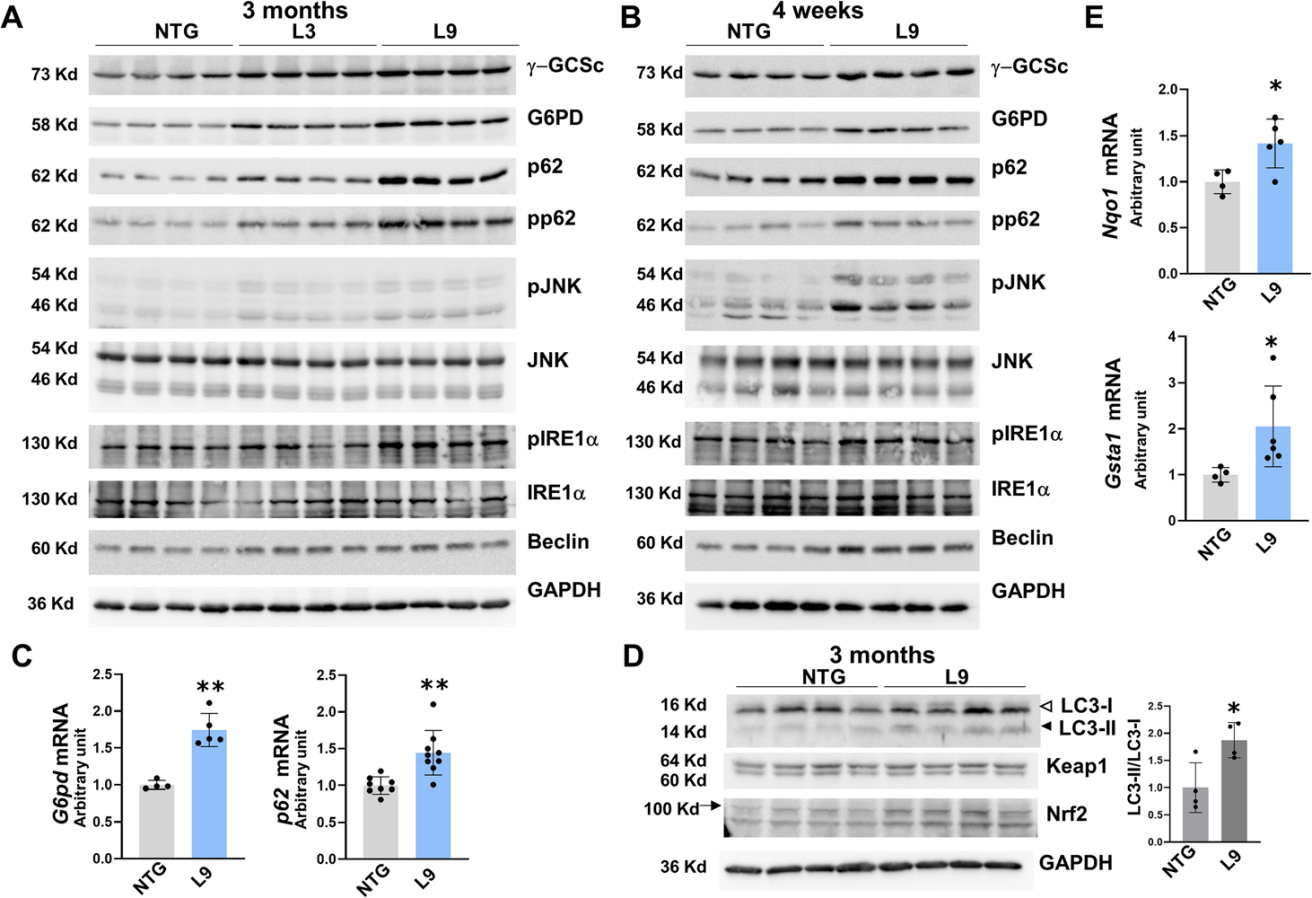
Analysis of expression of stress-induced molecules. **a** Western analysis of the hearts of 3-month old male L3 and L9 mice. **b** Western analysis of the hearts of 4-week old male L9 mice. **c** Quantitative RT-PCR analysis of *G6pd* and *p62* in the hearts of 3-month old male L9 mice. The results are presented as the means ± SD. Significance was assessed with unpaired *t-*test (*n* = 4 ~ 9; **p < 0.01 vs. NTG). **d** Western analysis of the hearts of 3-month-old male L9 mice. The white and black arrowheads respectively indicate LC3-I and LC3-II. An arrow indicates Nrf2. The right panels show relative densitometric analysis of the western blot of LC3. The results are presented as the means ± SD. Significance was assessed with unpaired *t-*test (*n* = 4; *p < 0.05 vs. NTG). **a**, **b** and **d**: Whole cell extracts were examined with the indicated antibodies. **e** Quantitative RT-PCR analysis of *Nqo1* and *Gsta1* in the hearts of 3-month old male L9 mice. The results are presented as the means ± SD. Significance was assessed with unpaired *t-*test (*n* = 4 ~ 6; *p < 0.05 vs. NTG). **a–e** Similar results were obtained in at least two independent experiments

Next, we examined the NADPH-to-NADP^+^ ratio in L9 hearts. However, the ratios in the L9 hearts were comparable to those observed in the NTG hearts (Fig. 4f). Since NADPH is produced from NADP^+^, primarily through the PPP, we further examined the expression of G6PD (the rate-limiting enzyme of the PPP) in transgenic hearts. The protein expression of G6PD was markedly upregulated in transgenic hearts compared with NTG hearts (L3: by 2.1-fold, L9: by 3.7-fold; Fig. 5a). Consistent with this finding, *G6pd* mRNA expression was 1.7 times higher in L9 hearts than in NTG hearts (Fig. 5c).

Thus, the GSH-to-GSSG ratio, GR activity and G6PD expression were elevated in transgenic hearts, but the ratio of NADPH to NADP^+^ was similar to that observed in NTG hearts. Since NADPH is an important electron source, it may have been consumed by redox couples, including glutathione systems.

GSH is also produced through de novo synthesis, which is mediated by two ATP-dependent ligases [56]. We examined the expression of the gamma-glutamylcysteine synthetase heavy subunit (γ-GCSc), a rate-limiting enzyme for de novo GSH production. γ-GCSc expression was 4.2 times higher in L9 hearts and 1.8 times higher in L3 hearts than that observed in NTG hearts (Fig. 5a, Additional file 8 A). The expression in 4-week old L9 mouse hearts was 1.6 times higher than that observed in NTG mouse hearts (Fig. 5b, Additional file 8 B). By contrast, γ-GCSc expression in 3-month old αMHC/miR-145TG mouse hearts was similar to that observed in NTG mouse hearts (Additional file 4 C), suggesting that miR-143-dependent acceleration of de novo GSH production occurred in αMHC/miR-143/145TG hearts.

### p62 expression is increased in transgenic hearts

Although p62 is a hub molecule for a variety of signaling processes, such as autophagy, stress responses and detoxification [22, 23], there is growing evidence that it is also crucial in GSH synthesis and glycolysis [20, 21]. We next examined the expression of p62 and found that it was markedly (8.6 times) higher in L9 hearts than that observed in NTG hearts (Fig. 5a, Additional file 8 A). Notably, the expressions of both G6PD and p62 were already higher in the hearts of L9 mice than in the hearts of NTG mice at 4 weeks of age (G6PD: 1.9 times higher, p62: 2.5 times higher) and were significantly higher in L9 hearts than in L3 hearts (G6PD: 1.7 times higher, p62: 3.3 times higher; Fig. 5a and b, Additional file 8 A and B).

Furthermore, *p62* mRNA expression was also increased in the hearts of L9 mice (Fig. 5c). Because JNK and IRE1α/JNK signaling have been shown to augment p62 expression [24–27], we next examined whether JNK and IRE1α were activated in transgenic hearts. Phosphorylation of JNK was significantly enhanced in L9 hearts (2.8 times higher compared to NTG hearts) and, to a lesser extent, in L3 hearts (1.6 times higher compared to NTG hearts; Fig. 5a, Additional file 8 A). Notably, phosphorylation of JNK was obviously higher in the hearts of 4-week old L9 mice (Fig. 5b, Additional file 8 B). Significantly (1.9 times) greater IRE1α phosphorylation was also detected in the hearts of L9 mice than in the hearts of NTG mice at 3 months of age, but this difference was not observed in mice at 4 weeks of age (Fig. 5a and b, Additional file 8 A and B).

Thus, JNK phosphorylation is likely to be associated with p62 expression in the transgenic hearts. However, IRE1α signaling does not appear to be a prerequisite for p62 expression, even though it may promote p62 expression. As a reductive state in the ER primarily induces stress by compromising disulfide bond formation, upregulation of p62 likely promotes IRE1α phosphorylation, and vice versa. Interestingly, the expression of Beclin, a regulator of autophagy downstream of JNK signaling [57], was also upregulated in transgenic hearts compared with that observed in NTG hearts (L3: by 1.3 times, L9: by 1.6 times; Fig. 5a and Additional file 8 A).

Since p62 accumulates upon inhibition of autophagy [22, 23], we investigated the conversion of LC3I to LC3II. This conversion was observed to be 1.9 times greater in L9 hearts than in NTG hearts (Fig. 5d). These data suggest that autophagy is facilitated in L9 hearts, which is also supported by the elevated Beclin expression (Fig. 5a).

Furthermore, we examined the expression of Keap1, an adaptor of the Cul3-ubiquitin E3 ligase complex responsible for Nrf2 that accumulates with p62 when autophagy is disturbed [58]. Its protein expression was not higher in L9 hearts (Fig. 5d). Collectively, these findings indicate that p62 was likely upregulated at the mRNA level rather than as a result of autophagy inhibition.

Nrf2 and p62 mutually enhance each another: Nrf2 signaling upregulates p62 expression [29], and p62 phosphorylation stabilizes the Nrf2 protein [30]. Nrf2 expression in L9 hearts was 1.5 times higher than that observed in NTG hearts (Fig. 5d) and phosphorylated p62 levels were higher in transgenic hearts than in NTG hearts (L3: 1.8 times higher, L9: 4.2 times higher; Fig. 5a and Additional file 8 A). These findings were consistent with the observation that the mRNA expressions of the Nrf2 targets, NAD(P)H quinone dehydrogenase 1 (*Nqo1*) and glutathione S-transferase alpha 1(*Gsta1*), were also augmented (Fig. 5e).

A similar pattern of upregulation of these signaling molecules was observed in L19 hearts (Additional file 5). However, the expressions of γ-GCSc, G6PD and p62 were not augmented in the hearts of αMHC/miR-145TG mice (Additional file 4 C; relative densitometric graphs corresponding to the western blots in Fig. 5a, b and d are shown in Additional file 8 A–C).

## Discussion

We established transgenic mice that expressed miR-143 at a high level in cardiomyocytes and exhibited a dilated cardiomyopathy-like phenotype. We further evaluated the protein expression of 5 miR-143 targets (HK2, ERK5, IGF1R, IGFBP5 and ORP8) that have been shown to be involved in cardiomyopathy or cardiac remodeling. Only HK2 expression was drastically suppressed in αMHC/miR-143/145TG mice. Furthermore, HK2 expression showed an inverse relationship to miR-143 expression in these mice.

Thus far, the validation of target genes for miRNAs has primarily been performed in cultured cells. Our findings strongly indicate that the validation of miRNAs in living animals is indispensable for evaluating their bona fide activity.

However, a significant difference in HK activity between αMHC/miR-143/145TG and NTG mice was not observed. The expression of HK1, another predominant HK isotype in the heart, did not increase by complementation. HK2 is under strong allosteric regulation by G6P in vivo [59], but the reason behind the discrepancy between in vitro HK activity and in vivo HK2 expression remains unclear.

Furthermore, the ATP content in the transgenic hearts was similar to that observed in the NTG hearts. This result may be explained by a marginal reduction of in vitro HK activity or by the metabolic substrate preference of the heart. In resting hearts, 60–90% of the acetyl-CoA that enters the tricarboxylic acid cycle comes from β-oxidation of free fatty acids, while 10–40% comes from the oxidation of pyruvate, which is derived in almost equal amounts from glycolysis and lactate oxidation [60].

Our current findings indicate that downregulation of HK2 expression is not crucial for the pathogenesis of the αMHC/miR-143/145TG phenotype. This finding is consistent with previous reports that heterozygous HK2-deficient mice display no overt cardiac phenotypes at baseline, although their hearts are more susceptible to ischemia or reperfusion injury after coronary ligation and pressure overload than those of wild-type mice [11–13]. However, experiments on mice in which the endogenous *Hk2* gene lacks the binding sequence for miR-143 are necessary to confirm our conclusion.

The conversion of GSSG to GSH requires NADPH, which is primarily supplied through the PPP. HK2 is a dominant supplier of G6P, which is the substrate for the first step of the PPP. It was therefore surprising that the glutathione redox state in αMHC/miR-143/145TG hearts was reductive rather than oxidative. Meanwhile, our findings indicate enhanced GR activity and G6PD and γ-GCSc expression in the transgenic hearts, indicating that both the recycling and de novo biosynthesis of GSH were facilitated.

Although there is widespread consensus that oxidative stress elicits diverse pathophysiological processes, including cardiovascular complications, antioxidant supplementation has failed to hinder the progression of related disorders [17]. However, the involvement of reductive stress in a variety of diseases has received considerable attention in recent years [14–16]. In particular, G6PD, a rate-limiting enzyme for the PPP, has been proven to be a crucial molecule in reductive stress processes [18].

Valencia et al. reported that p62 influences metabolic pathways by controlling glycolysis and cellular redox processes in fibroblasts, including NADPH production and GSH synthesis [21]. p62 also plays pivotal roles in the production of GSH and the promotion of tumor formation [20]. Notably, the protein levels of p62 and G6PD were already elevated in the hearts of L9 mice at 4 weeks of age. In addition, we found greater phosphorylation of JNK and IRE1α and activation of Nrf2 signaling in transgenic hearts. These processes have been known to activate p62 [24–27, 29]. We postulate that a reductive redox shift may be involved in the pathogenesis of the αMHC/miR-143/145TG phenotype.

Our cDNA microarray findings concurred with our p62 findings. They also indicate markedly higher expression of genes related to glucose metabolism in transgenic hearts than in NTG hearts. Consistent with this finding, electron microscopic examination results showed substantially more glycogen granules in L9 hearts than in NTG hearts (Additional file 9). Furthermore, the number of granules was already elevated in L9 mice at 4 weeks of age, indicating that dysregulation of glucose metabolism precedes cardiac remodeling.

However, the NADPH-to-NADP^+^ ratio was not elevated in αMHC/miR-143/145TG mice despite the increased expression of G6PD and p62. We do not have an explanation for this discrepancy. NADPH may have been consumed by redox couples, including glutathione systems. In addition, such a significant increase in the NADPH-to-NADP^+^ ratio may require an enhancement in HK2 activity, although previous studies of animal models for reductive stress did not reveal the activity or expression of HK2. Further studies are needed to assess this possibility.

Our findings suggest that autophagy was facilitated in the hearts of L9 mice. This result is unexpected, because previous studies demonstrated that miR-143 suppressed autophagy through downregulation of ATG2B [61, 62]. Since those investigators used human cancer cell lines for the miR-143-transfection assays, the discrepancy may be due to differences in cellular context or animal species. Further investigation should be performed.

The redox state of a redox couple is defined by the half-cell reduction potential and the reducing capacity of that couple. As the concentration of GSH is far higher (millimolar levels) than the concentrations of most other redox active compounds, GSH is regarded as the principal redox buffer in cells [60]. Thus, we consider the redox state of the hearts of αMHC/miR-143/145TG mice to be reductive.

Mutations in αB-crystallin provoke myopathy and cardiomyopathy, which are characterized by protein misfolding and the formation of large cytoplasmic aggregates [18, 63]. Additionally, accumulation of mutant lamin aggregates can promote p62 expression and elicit reductive stress in human *LMNA*-mutant myopathy and corresponding *Drosophila* models [19]. Reductive stress has also been detected in healthy individuals with a predisposition to Alzheimer’s disease, which is considered to be caused by protein aggregation [64]. Although further investigation is required, the aggregation of misfolded proteins may trigger the pathogenesis observed here, which is likely aggravated by reductive redox shift-induced IRE1α signaling.

**Fig. 6:**
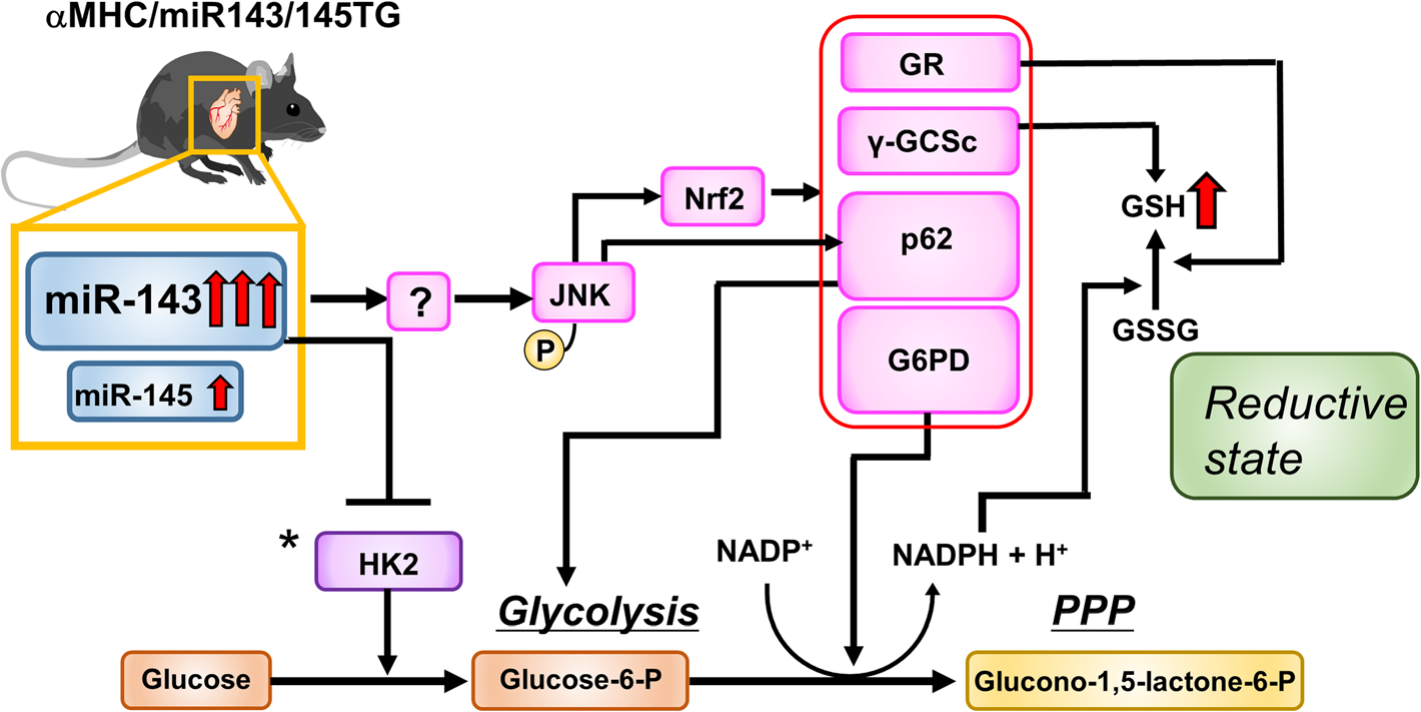
Schematic depiction of the signaling cascade in αMHC/miR-143/145TG mice. A hypothetical signaling model for a reductive state in αMHC/miR-143/145TG mice is shown. Our data indicate that the overexpression of miR-143 plays a pivotal role in the pathogenesis of the αMHC/miR-143/145TG phenotype, but the key targets for miR-143 triggering this process have not been identified and are shown with a question mark. *: Although the expression of HK2 was suppressed in transgenic hearts, in vitro HK activity was comparable in the L9 and NTG mouse hearts. Since the expression levels of GR, γ-GCSC, p62 and G6PD are controlled by Nrf2, these four molecules are surrounded by a red box. A detailed explanation is given in the Discussion section

Given all these findings, we propose that the overexpression of miR-143 in cardiomyocytes in the mouse lines generated in this study initially promotes the phosphorylation of JNK and the expression of Nrf2, G6PD and p62. Sustained activation of these signaling pathways may induce a reductive redox shift, resulting in cardiomyopathy (Fig. 6).

We have not yet identified the targets of miR-143 and miR-145 that are responsible for the pathogenesis of the phenotype observed in αMHC/miR-143/145TG mice. We are planning to investigate the other target candidates for miR-143 detected in our cDNA microarray analysis (e.g., *elk-1* and *adducin-3*). Malkovich et al. also reported a downregulation of HK2 expression in the hearts of αMHC/miR-143 transgenic mice that was not associated with any deleterious phenotype [5]. Thus, even though transgenic miR-145 expression was quite low in the αMHC/miR-143/145TG hearts in this study, miR-145 may also be involved in the pathogenetic mechanism in cooperation with miR-143.

## Conclusions

Transgenic expression of miR-143/145 in mice cardiomyocytes induced a dilated cardiomyopathy-like phenotype with a reductive redox shift. Unfortunately, because the molecular pathogenesis of dilated cardiomyopathy is diverse and complicated, the impact of treatment is currently far from satisfactory. We believe that our unique mouse lines will be useful for elucidating the mechanisms of at least some types of dilated cardiomyopathy.

## Supplementary information

**Additional file 1.** List of primer sequences. F = forward primer, R = reverse primer

**Additional file 2.** List of antibodies for Western blot analysis. The first and the second antibodies were generally diluted at a dilution of 1:1000, and 1:20000, respectively, but anti-GAPDH-HRP antibody was diluted at a dilution of 1:2000.

**Additional file 3. **Examination of the aged αMHC/miR-143/145 L3 TG mice. Hematoxylin & Eosin-stained hearts of 6-month-old male NTG mouse (A, B), 6-month-old male L3 mouse (C), and the male L3 mouse that died at 8 months of age (D). (E) Heart weight corrected for tibia length (upper panel) or body weight (lower panel) of 6-month-old L3 male mice. Results represent the mean ± SD with scattered blots. Unpaired *t*-test (*n* = 11. *P < 0.05 vs. NTG; **P < 0.01 vs. NTG).

**Additional file 4. **Establishment and analysis of αMHC/miR-145TG mice. (A) Construction of the injected fragment. An approximate 6.3 kb Bam HI fragment containing the pri-miR-145 gene was used. (B) qRT-PCR analysis of miR-143 and miR-145 in the hearts of 3-month-old male αMHC/miR-145TG. Bars present mean ± SD. Unpaired *t*-test (*n* = 3 ~ 4. *P < 0.05 vs. NTG; **P < 0.01 vs. NTG). (C) Western blot analysis of the hearts of 3-month-old male αMHC/miR-145TG mice. Whole cell extracts were examined with antibodies indicated. Relative densitometric analysis of the western blots is shown in the right panels. Bars present mean ± SD. Unpaired *t*-test (n = 3 ~ 4; *P < 0.05 vs. NTG, **P < 0.01 vs. NTG). B, C; Similar results were obtained in at least two independent experiments.

**Additional file 5. **Western blot analysis of the hearts of 3-month-old male L19 mice. Whole cell extracts were examined with antibodies indicated. An arrow head indicates HK2 band. Relative densitometric analysis of the western blots is shown in the right panels. Bars present mean ± SD. Unpaired *t*-test (*n* = 4; *P < 0.05 vs. NTG, **P < 0.01 vs. NTG). Similar results were obtained in at least two independent experiments.

**Additional file 6. **Western blot analysis of the target molecules for miR-143. Whole cell extracts of the hearts of 3-month-old male αMHC/miR-143/145TG mice were examined with antibodies indicated. Relative densitometric analysis of the western blots is shown in the right panels. Bars present mean ± SD. Unpaired *t*-test (n = 4). Similar results were obtained in at least two independent experiments.

**Additional file 7.** Establishment and analysis of αMHC/HK2TG and αMHC/ miR-143/145/HK2TG mice. (A) Construction of the injected fragment for αMHC/HK2TG mice. About 8.8 kb Bam HI fragment containing the human HK2 cDNA was used. (B) Western blot analysis of the hearts of 2-month-old male αMHC/HK2TG mice. The size of human exogenous HK2 bands is larger than that of mouse endogenous one. Whole cell extracts were examined with antibodies indicated. Similar results were obtained in at least two independent experiments. Kaplan Meier survival analysis of αMHC/miR-143/145/HK2TG mice #1 (C) and #2 (D). Data were analyzed using long-rank test.

**Additional file 8. **Relative densitometric analysis of the western blots. (A) Analysis of the western blots (Fig. 5a). Bars present mean ± SD. One-way ANOVA followed by a post hoc Tukey test (n = 4: *P < 0.05 vs. NTG; **P < 0.01 vs. NTG; **†***P* < 0.05 vs. L3; **††** *P* < 0.01 vs. L3). (B) Analysis of the western blots (Fig. 5b). Bars present mean ± SD. Unpaired *t*-test (n = 4; *P < 0.05 vs. NTG; **P < 0.01 vs. NTG). (C) Analysis of the western blots (Fig. 5d). Bars present mean ± SD. Unpaired *t*-test (n = 4; *P < 0.05 vs. NTG). A-C; Similar results were obtained in at least two independent experiments.

**Additional file 9.** Electron microscopic analysis of the hearts of female L9 mice. 3-month-old NTG (A) and L9 mouse (B). 4-week-old NTG (C) and L9 mouse (D). White arrows indicate the glycogen granules.

## Data Availability

The microarray data were deposited in NCBI GEO under accession number GSE112355.

## Abbreviations

miRNA: MicroRNA
αMHC: Alpha-myosin heavy chain
PPP: Pentose phosphate pathway
EDTA: Ethylenediaminetetraacetic acid
NADPH: Reduced nicotinamide adenine dinucleotide phosphate
NADP^+^: Oxidized nicotinamide adenine dinucleotide phosphate
HK2: Hexokinase 2
HK1: Hexokinase 1
G6P: Glucose-6-phosphate
G6PD: Glucose-6-phosphate dehydrogenase
Nrf2: Nuclear factor erythroid-2 related factor 2
JNK: Jun N-terminal kinase
IRE1α: Inositol-requiring enzyme 1 alpha
ER: Endoplasmic reticulum
γ-GCSc: Gamma-glutamylcysteine synthetase heavy subunit
GSSG: Glutathione-S-S-glutathione, reduced glutathione
GSH: Glutathione-SH, oxidized glutathione
TBARS: Thiobarbituric acid reactive substances
Anp: Atrial natriuretic peptide
Bnp: Brain natriuretic peptide
β-Mhc: Beta-myosin heavy chain
Serca2A: Sarco-endoplasmic reticulum calcium adenosine triphosphatase-2A
NQO1: NAD(P) H quinone dehydrogenase 1
Gsta1: Glutathione S-transferase alpha 1
ERK5: Extracellular signal-regulated protein kinase 5
IGF1R: Insulin-like growth factor 1 receptor
IGFBP5: Insulin-like growth factor-binding protein 5
ORP8: Oxysterol-binding protein-related protein 8
ATP: Adenosine triphosphate
PBS: Phosphate-buffered saline
GR: Glutathione reductase
