# Supplementary figures and images for: Forced expression of miR-143 and -145 in cardiomyocytes induces cardiomyopathy with a reductive redox shift
Source: Cell Mol Biol Lett. 2020 Aug 24;25:40. doi: 10.1186/s11658-020-00232-x (PMC7444248; doi:10.1186/s11658-020-00232-x)

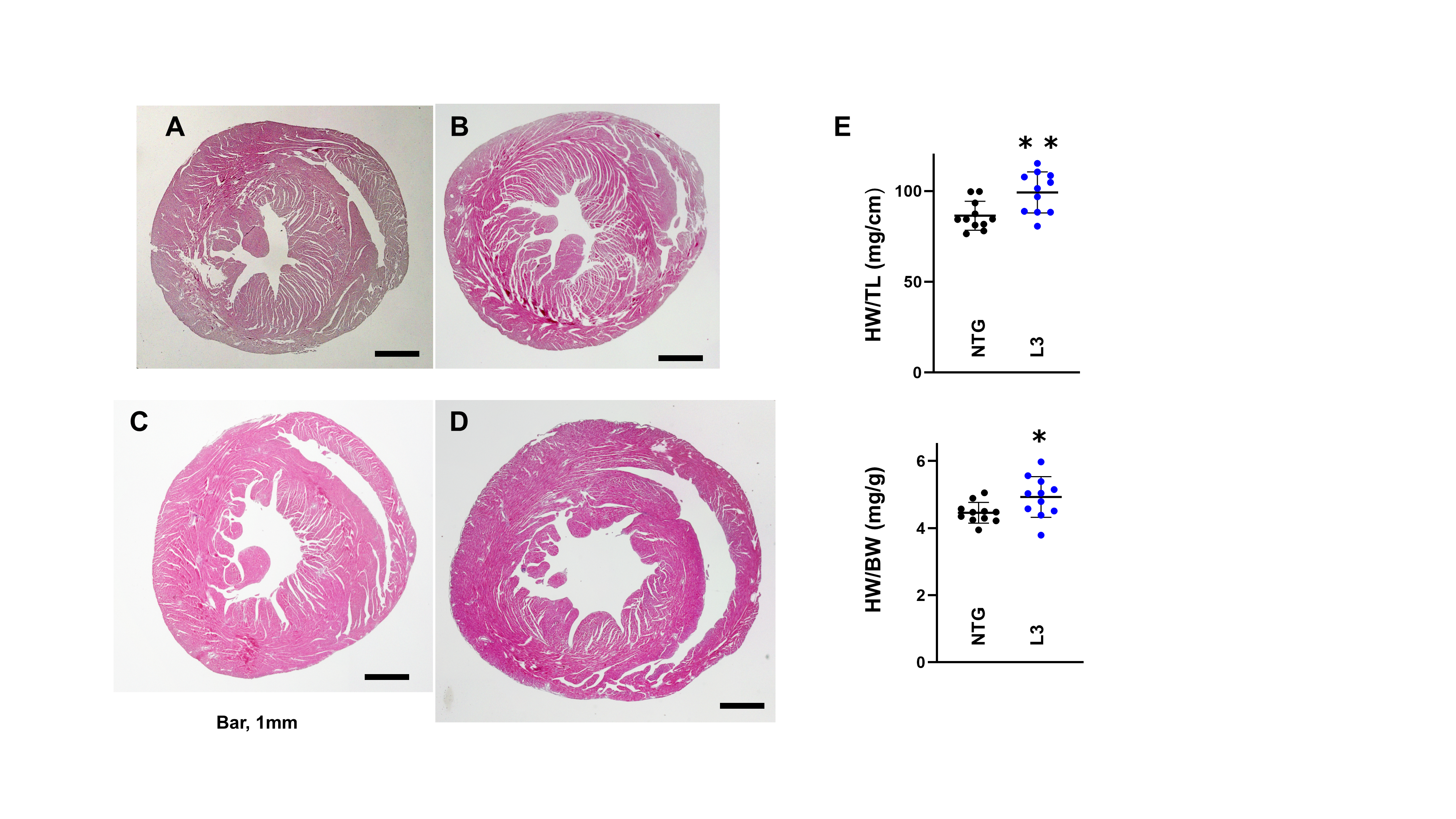

Supplement: Supplementary file 3 — Additional file 3. Examination of the aged αMHC/miR-143/145 L3 TG mice. Hematoxylin & Eosin-stained hearts of 6-month-old male NTG mouse (A, B), 6-month-old male L3 mouse (C), and the male L3 mouse that died at 8 months of age (D). (E) Heart weight corrected for tibia length (upper panel) or body weight (lower panel) of 6-month-old L3 male mice. Results represent the mean ± SD with scattered blots. Unpaired t-test (n = 11. *P < 0.05 vs. NTG; **P < 0.01 vs. NTG). [file 11658_2020_232_MOESM3_ESM.tif]

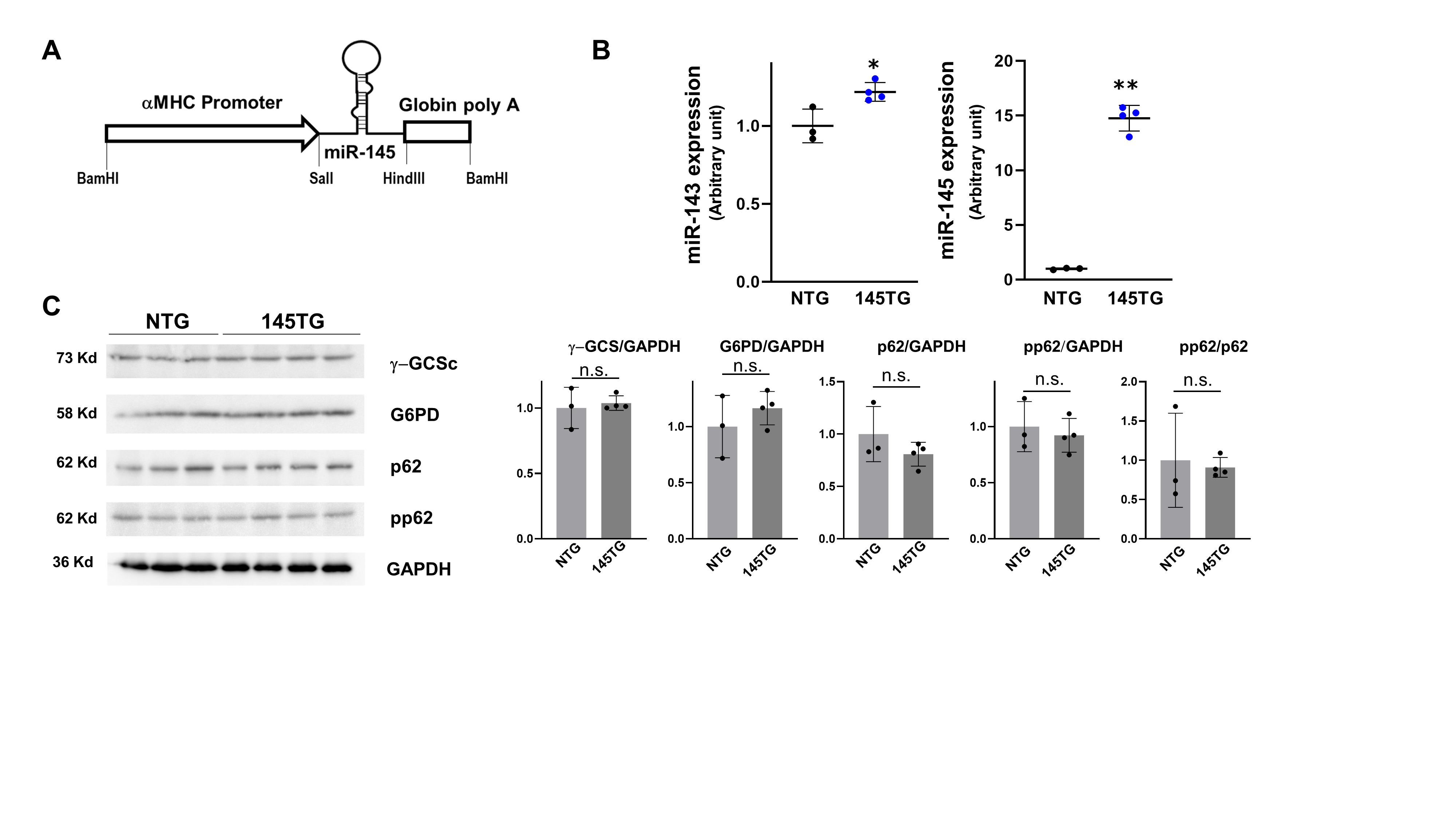

Supplement: Supplementary file 4 — Additional file 4. Establishment and analysis of αMHC/miR-145TG mice. (A) Construction of the injected fragment. An approximate 6.3 kb Bam HI fragment containing the pri-miR-145 gene was used. (B) qRT-PCR analysis of miR-143 and miR-145 in the hearts of 3-month-old male αMHC/miR-145TG. Bars present mean ± SD. Unpaired t-test (n = 3 ~ 4. *P < 0.05 vs. NTG; **P < 0.01 vs. NTG). (C) Western blot analysis of the hearts of 3-month-old male αMHC/miR-145TG mice. Whole cell extracts were examined with antibodies indicated. Relative densitometric analysis of the western blots is shown in the right panels. Bars present mean ± SD. Unpaired t-test (n = 3 ~ 4; *P < 0.05 vs. NTG, **P < 0.01 vs. NTG). B, C; Similar results were obtained in at least two independent experiments. [file 11658_2020_232_MOESM4_ESM.tif]

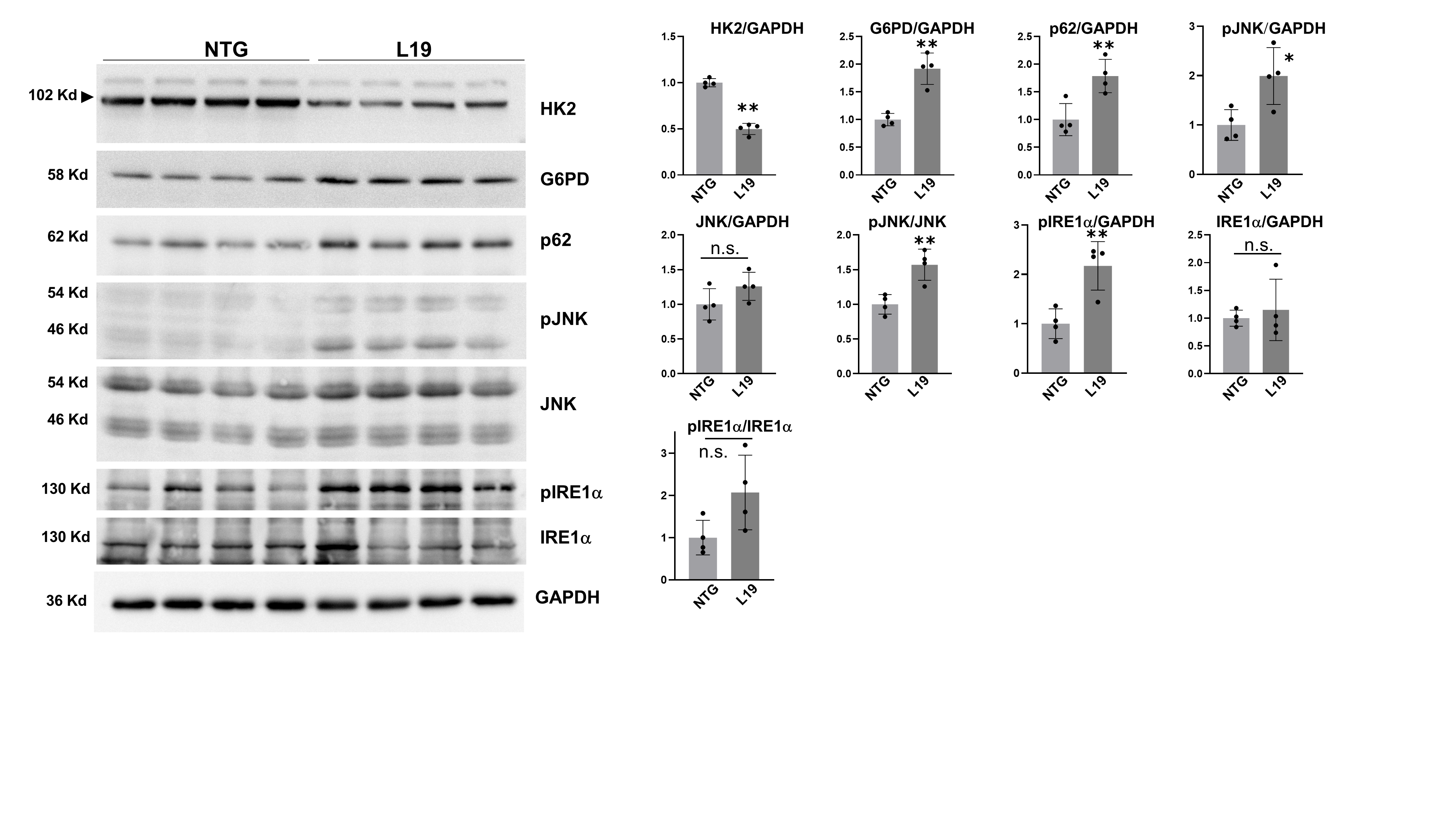

Supplement: Supplementary file 5 — Additional file 5. Western blot analysis of the hearts of 3-month-old male L19 mice. Whole cell extracts were examined with antibodies indicated. An arrow head indicates HK2 band. Relative densitometric analysis of the western blots is shown in the right panels. Bars present mean ± SD. Unpaired t-test (n = 4; *P < 0.05 vs. NTG, **P < 0.01 vs. NTG). Similar results were obtained in at least two independent experiments. [file 11658_2020_232_MOESM5_ESM.tif]

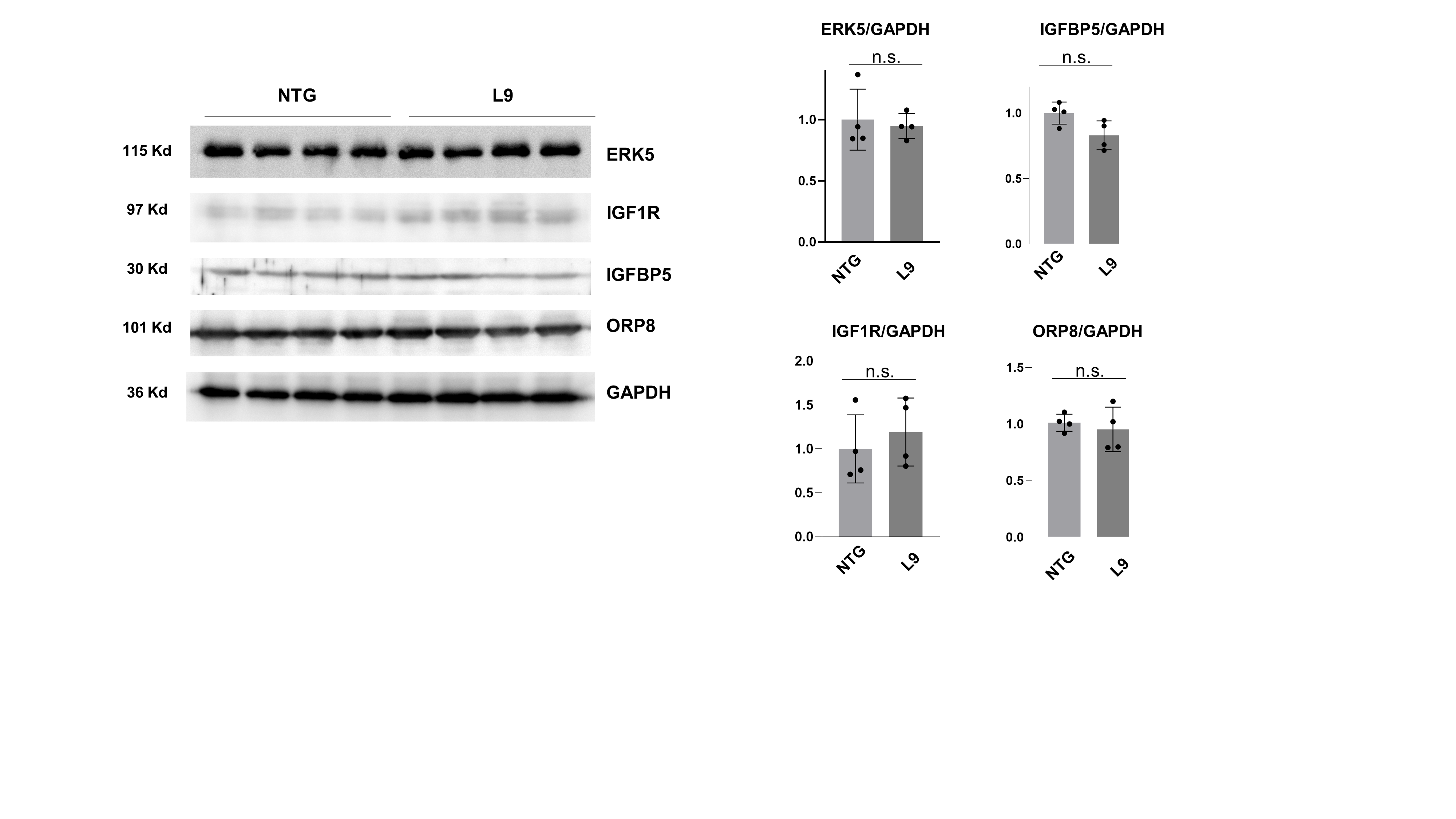

Supplement: Supplementary file 6 — Additional file 6. Western blot analysis of the target molecules for miR-143. Whole cell extracts of the hearts of 3-month-old male αMHC/miR-143/145TG mice were examined with antibodies indicated. Relative densitometric analysis of the western blots is shown in the right panels. Bars present mean ± SD. Unpaired t-test (n = 4). Similar results were obtained in at least two independent experiments. [file 11658_2020_232_MOESM6_ESM.tif]

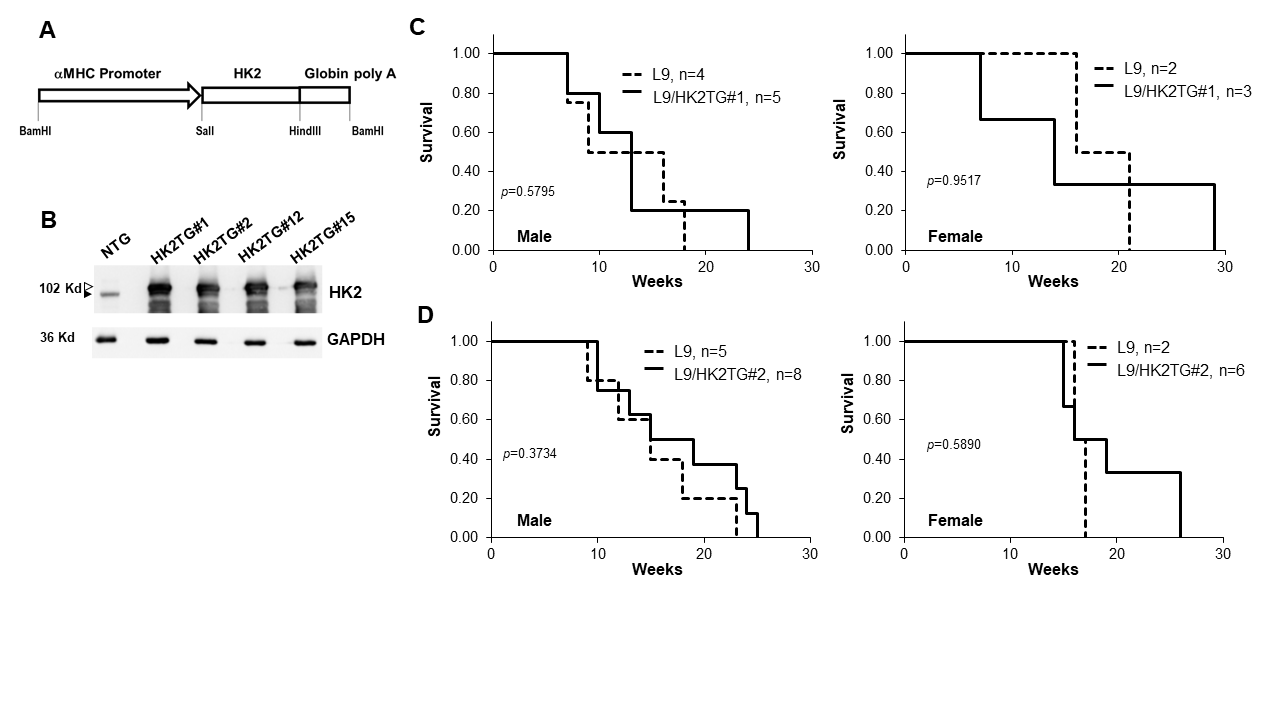

Supplement: Supplementary file 7 — Additional file 7. Establishment and analysis of αMHC/HK2TG and αMHC/ miR-143/145/HK2TG mice. (A) Construction of the injected fragment for αMHC/HK2TG mice. About 8.8 kb Bam HI fragment containing the human HK2 cDNA was used. (B) Western blot analysis of the hearts of 2-month-old male αMHC/HK2TG mice. The size of human exogenous HK2 bands is larger than that of mouse endogenous one. Whole cell extracts were examined with antibodies indicated. Similar results were obtained in at least two independent experiments. Kaplan Meier survival analysis of αMHC/miR-143/145/HK2TG mice #1 (C) and #2 (D). Data were analyzed using long-rank test. [file 11658_2020_232_MOESM7_ESM.tif]

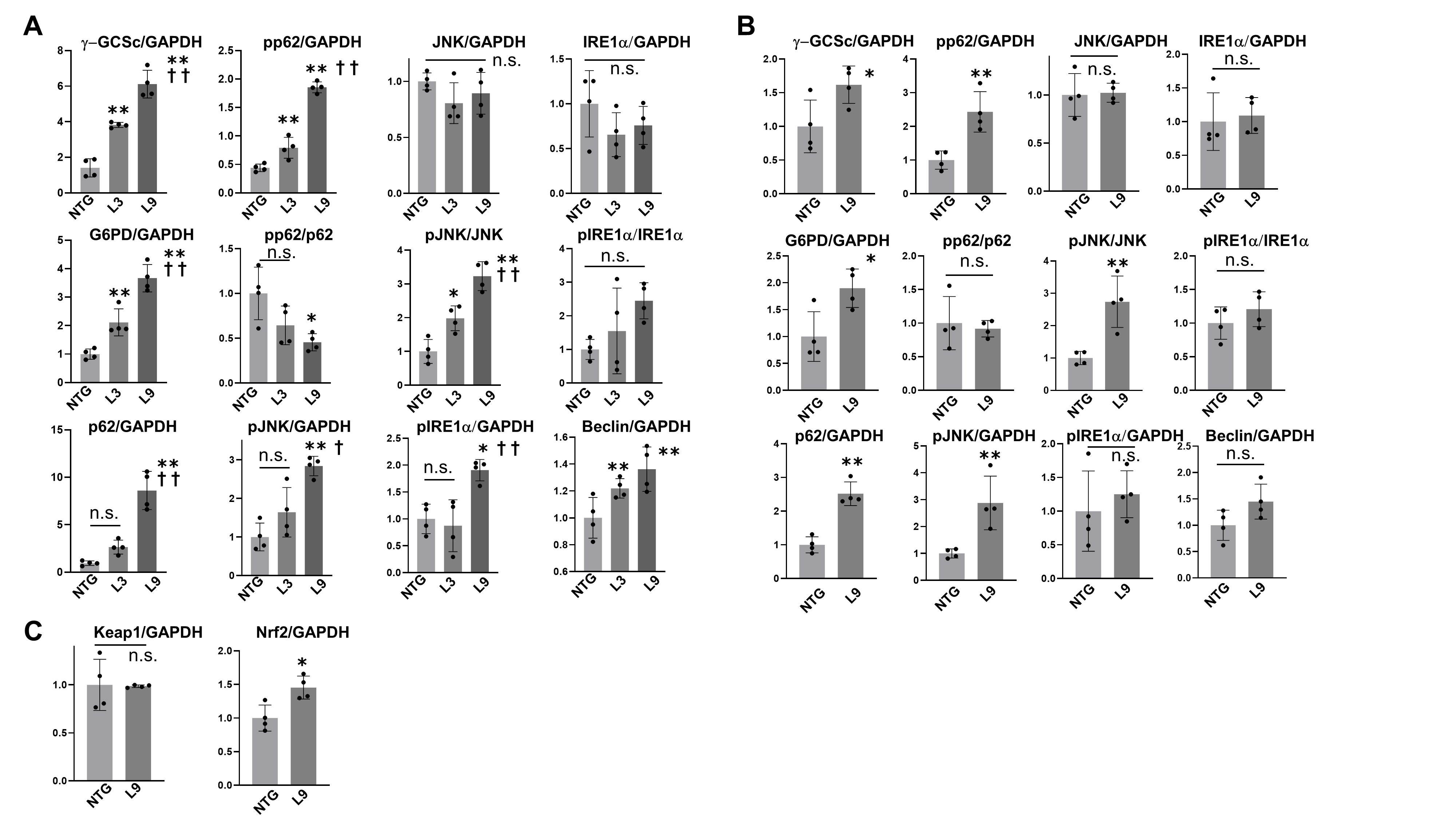

Supplement: Supplementary file 8 — Additional file 8. Relative densitometric analysis of the western blots. (A) Analysis of the western blots (Fig. 5a). Bars present mean ± SD. One-way ANOVA followed by a post hoc Tukey test (n = 4: *P < 0.05 vs. NTG; **P < 0.01 vs. NTG; †P < 0.05 vs. L3; †† P < 0.01 vs. L3). (B) Analysis of the western blots (Fig. 5b). Bars present mean ± SD. Unpaired t-test (n = 4; *P < 0.05 vs. NTG; **P < 0.01 vs. NTG). (C) Analysis of the western blots (Fig. 5d). Bars present mean ± SD. Unpaired t-test (n = 4; *P < 0.05 vs. NTG). A-C; Similar results were obtained in at least two independent experiments. [file 11658_2020_232_MOESM8_ESM.tif]

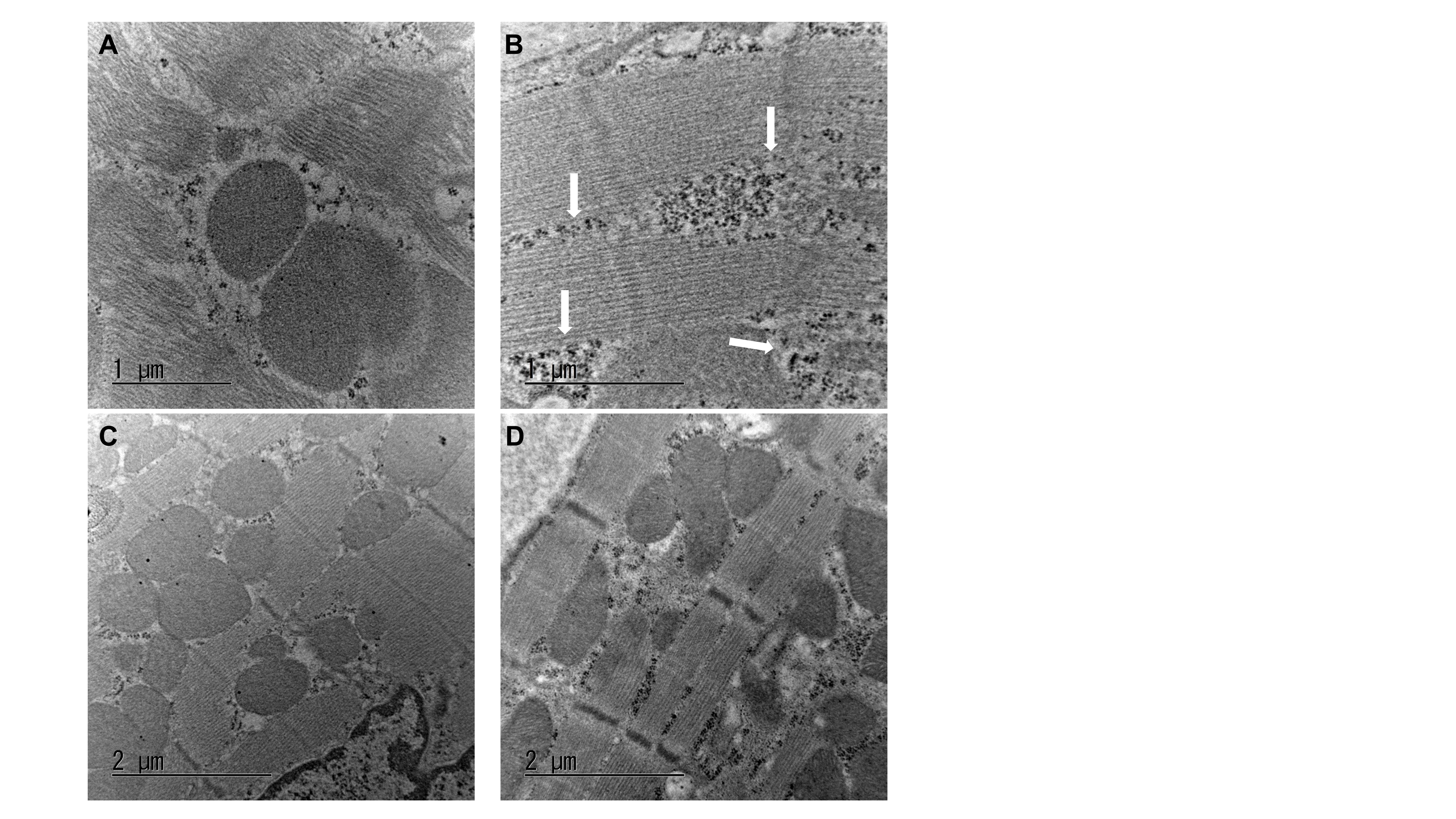

Supplement: Supplementary file 9 — Additional file 9. Electron microscopic analysis of the hearts of female L9 mice. 3-month-old NTG (A) and L9 mouse (B). 4-week-old NTG (C) and L9 mouse (D). White arrows indicate the glycogen granules. [file 11658_2020_232_MOESM9_ESM.tif]
